# Blocking Tryptophan Catabolism Reduces Triple-Negative Breast Cancer Invasive Capacity

**DOI:** 10.1158/2767-9764.CRC-24-0272

**Published:** 2024-10-16

**Authors:** Li-Wei Kuo, Lyndsey S. Crump, Kathleen O’Neill, Michelle M. Williams, Jessica L. Christenson, Nicole S. Spoelstra, Micaela Kalani Roy, Amy Argabright, Julie A. Reisz, Angelo D’Alessandro, Meher P. Boorgula, Andrew Goodspeed, Mike Bickerdike, Benjamin G. Bitler, Jennifer K. Richer

**Affiliations:** 1 Cancer Biology Training Program, University of Colorado Anschutz Medical Campus, Aurora, Colorado.; 2 Department of Pathology, University of Colorado Anschutz Medical Campus, Aurora, Colorado.; 3 Department of Biochemistry and Molecular Genetics, University of Colorado Anschutz Medical Campus, Aurora, Colorado.; 4 University of Colorado Cancer Center, University of Colorado Anschutz Medical Campus, Aurora, Colorado.; 5 Department of Biomedical Informatics, University of Colorado Anschutz Medical Campus, Aurora, Colorado.; 6 Antido Therapeutics, Melbourne, Australia.; 7 Division of Reproductive Sciences, Department of Obstetrics and Gynecology, University of Colorado Anschutz Medical Campus, Aurora, Colorado.

## Abstract

Anchorage-independent triple-negative breast cancer (TNBC) cells exhibit elevated levels of the tryptophan (TRP)-catabolizing enzyme tryptophan 2,3-dioxygenase 2 (TDO2) compared with the same cells grown under two-dimensional culture conditions. Tracing of ^13^C_11_-TRP demonstrated that anchorage-independent culture and/or inflammatory cytokines that activate NF-κB increase TRP catabolism and production of downstream catabolites such as kynurenine, which activate the aryl hydrocarbon receptor (AhR). TDO2 expression is heterogeneous within TNBC cell lines. To determine the function of TDO2, both pharmacologic inhibition and genetic manipulation were conducted. TDO2 knockdown revealed a compensatory increase in indoleamine 2,3-dioxygenase 1 (IDO1), a nonhomologous TRP-catabolizing enzyme, indicating that dual inhibition of these two enzymes is necessary to reliably block TRP catabolism. Thus, we tested a newly developed TDO2/IDO1 dual inhibitor, AT-0174, and found that it effectively inhibits TNBC TRP catabolism. Furthermore, AT-0174 treatment or AhR inhibitor significantly decreased TNBC anchorage-independent survival, invasive capacity, and expression of mesenchymal genes and proteins, whereas exogenous kynurenine increased invasion through AhR-mediated zinc finger E-box–binding homeobox 1 (ZEB1) expression. Thus, dual inhibition of TDO2/IDO1 may prove efficacious against TNBC progression.

**Significance:**

TDO2 is more highly expressed than the nonhomologous TRP-catabolizing enzyme IDO1 in TNBC. We find that TDO2 knockdown can lead to a compensatory increase in IDO1. Therefore, we tested a newly developed TDO2/IDO1 dual inhibitor and found that it decreases TRP catabolism, anchorage-independent survival, and invasive capacity.

## Introduction

Triple-negative breast cancer (TNBC) has a high rate of recurrence as a metastatic disease within the first few years of diagnosis (1–3). Anchorage-independent survival (also known as anoikis resistance) is known to facilitate metastasis (4–7). We previously demonstrated that upregulation of the kynurenine (KYN) pathway for tryptophan (TRP) catabolism through NF-κB occurs in anoikis-resistant/anchorage-independent TNBC cells. Inflammatory cytokines, IL1β, and TNFα that activate NF-κB also support anchorage independence (8). Expression of tryptophan 2,3-dioxygenase 2 (TDO2), a rate-limiting enzyme in the KYN pathway, is increased in anoikis-resistant TNBC (9–11) and ovarian cancer cells (12) grown under anchorage-independent conditions compared with two-dimensional culture, and furthermore, anchorage-independent survival is dependent on TDO2 activity (13).

TDO2 and the nonhomologous enzymes indoleamine 2,3-dioxygenase 1 and 2 (IDO1 and IDO2) evolved separately to catabolize TRP to formyl-KYN and KYN. IDO1 is expressed in most tissues, as is IDO2, albeit at much lower levels (14). TDO2 is normally expressed in the brain and liver (14). However, TDO2 and IDO1 can be expressed in various cancers. KYN binds to the aryl hydrocarbon receptor (AhR) and promotes cancer cell survival and motility in human glioblastoma cells (15) and breast and ovarian cancer cells (8, 13). In addition to binding AhR in cancer cells, secreted KYN can bind to AhR in immune cells, resulting in the functional attenuation of cytotoxic T cells and expansion of regulatory T cells, to facilitate an immunosuppressive microenvironment (14–16). In publicly available primary breast cancer databases, high tumor *TDO2* correlates with poor overall survival (16). Although TDO2 mRNA and proteins are higher than those of IDO1 in TNBC, both enzymes can be expressed (8, 16), and we find this to be the case in ovarian cancer as well (12, 13).

Current small-molecule inhibitors of TDO2 or IDO1 are tryptophan mimetics (14). In clinical trials, IDO1 inhibitors such as epacadostat were combined with immune checkpoint blockade, such as anti-programmed death-ligand 1 (anti–PD-L1) or anti–programmed cell death protein 1 (anti-PD-1; refs. 17, 18); however, no significant improvement in tumor response was observed (19). Dual IDO1/TDO2 inhibitors are still under preclinical investigation and are currently in phase I clinical trials (20, 21). Although the mechanisms of resistance to selective TDO2 or IDO1 inhibitors are largely unexplored, resistance is hypothesized to arise from the compensatory potential of these enzymes that catabolize TRP (17). Thus, dual inhibition of TDO2 and IDO1 may be necessary to block TRP catabolism effectively.

Here, we examined TNBC response to inflammatory cytokines that increase endogenous TDO2 and measured flux through the TRP catabolism pathway by tracing stable isotope-labeled TRP in anchorage-independent versus standard two-dimensional culture, with or without inflammatory cytokines and with or without a new dual IDO1/TDO2 inhibitor. We find that knockdown (KD) of TDO2 can lead to a compensatory increase in IDO1, indicating the necessity for dual inhibition of both enzymes. We find that AT-0174, a new small-molecule dual TDO2/IDO1 inhibitor, significantly reduced TRP catabolites produced by TNBC cells and reduced anchorage-independent survival and invasion through a TDO2/KYN/AhR/zinc finger E-box binding homeobox 1 (ZEB1) axis. Collectively, our findings demonstrate that dual TDO2/IDO1 inhibition is a promising therapeutic avenue for the blockade of TRP catabolism to reduce TNBC progression to metastatic disease.

## Materials and Methods

### Cell culture

BT549 (No. HTB-122; RRID: CVCL_1092) was purchased from ATCC and cultured in RPMI 1640 medium supplemented with 10% FBS, 1% non-essential amino acids (NEAA), and 10 μg/mL insulin. MDA-MB-453 (No. HTB-131; RRID: CVCL_0418) was obtained from ATCC and cultured in DMEM with 10% FBS. SUM159PT (RRID: CVCL_5423) was obtained from the University of Colorado Cancer Center (UCCC) Cell Technology Shared Resource and grown in Ham’s F12 media with 5% FBS, 1% 4-(2-Hydroxyethyl)piperazine-1-ethane-sulfonic acid (HEPES), 10 µg/mL insulin, and 0.3% hydrocortisone. MDA-MB-231 (No. HTB-26; RRID: CVCL_0062) was purchased from ATCC and cultured in 5% FBS, 1% HEPES, 1% NEAA, 1% glutamine, 1% penicillin–streptomycin, and 10 µg/mL insulin. All cell lines were authenticated by short tandem repeat analysis and confirmed *Mycoplasma* negative in the UCCC Cell Technology Shared Resource before the study. Cells were last tested on May 2, 2024. For experiments in anchorage-independent culture (forced suspension condition), the culture plates were coated with 12 mg/mL of poly-2-hydroxyethyl methacrylate (poly-HEMA; Cat. # P3932, Sigma-Aldrich) and dried overnight before cell seeding.

### Single-cell RNA sequencing

A total of 5 × 10^5^ MDA-MB-453 cells were seeded in T25 flasks. After 24 hours, the cells were treated with either vehicle (water) or 10 ng/mL of IL1β + TNFα for 24 hours. A total of 3,000 cells were collected for single-cell RNA sequencing (scRNA-seq) with a read depth of 75,000 reads/cell by using the 10× Genomics platform (RRID: SCR_019326) and NovSeq 6000 system (Illumina) at the UCCC Genomics Shared Resource Facility (RRID: SCR_021984) according to 10× sample preparation guideline. For data analysis, Cell Ranger (v3.1.0; ref. 22) was used to process the FASTQ files to generate cell and gene count tables using unique molecule identifiers (UMI) with the GRCh38 genome (compiled by 10× Genomics, refdata-gex-GRCh38-2020-A). The Seurat (v4.0.4; ref. 23) pipeline was used for downstream quality control and analysis. Cell Ranger–filtered data were read into Seurat. Host genes were removed if identified in fewer than 10 cells. Cells were filtered out if they contained ≥50,000 UMI, ≤3,000 genes, ≥5,000 genes, or ≥15% of UMIs coming from mitochondria. The two samples were integrated for visualization only using Seurat with normalization by the sctransform with the difference between S and G_2_–M scores regressed out. The top 30 principal components were used to perform Uniform Manifold Approximation and Projection. Prior to differential expression and plotting gene expression, data were normalized by dividing gene counts by total counts per cell and multiplied by 10,000 followed by natural-log transformation. Raw and processed data are deposited in Gene Expression Omnibus (GEO; GSE237918). Individual analysis of the control cells was previously published (24).

### Bulk RNA sequencing

AT-0174 treatment set: 3 × 10^5^ BT549 cells were seeded in a six-well plate coated with 12 mg/mL poly-HEMA for forced suspension culture and followed by 10 µmol/L AT-0174 or vehicle (DMSO) treatment. BT549 sh*TDO2* set: 3 × 10^5^ BT549 TDO2-KD shRNA constructs and shScramble (shSCR) control cells were seeded in a six-well plate. MDA-MB-453 set: 5 × 10^5^ MDA-MB-453 cells were seeded in a six-well plate coated with/without 12 mg/mL poly-HEMA for forced suspension/regular cultures. All the conditions were conducted in biological triplicate. After 48 hours, the total RNAs were extracted using RNeasy Mini Kit (Cat. # 74104, QIAGEN). For library preparation and sequencing, RNA quality was verified using a High Sensitivity ScreenTape Assay on the TapeStation 2200 system (Agilent Technologies) and measured using a NanoDrop 1000 (Thermo Fisher Scientific). Library construction was performed using the Universal Plus mRNA Library Kit (NuGEN Technologies), and sequencing was performed on the NovaSeq 6000 instrument (Illumina) using paired-end sequencing (2 x 150 bp) by the UCCC Genomics Shared Resource (RRID: SCR_021984). For data analysis, RNA sequencing (RNA-seq) data were processed using the nf-core/rnaseq pipeline (version 3.12.0; ref. 25). Briefly, Illumina adapters were removed using Cutadapt (version 3.4; ref. 26) as part of the Trim Galore (0.6.7) package (https://doi.org/10.5281/zenodo.7598955). Reads were aligned using STAR (version 2.7.9a; ref. 27) to the human transcriptome (GRCh38, gene annotation from Ensembl release 104) and quantified using Salmon (version 1.10.1; ref. 28). Raw data with counts by gene were generated using tximport (https://doi.org/10.12688/f1000research.7563.1) on Salmon-quantified data. Normalized data were generated as counts per million (29). Differential expression was calculated between groups using the limma R package (30). Gene set enrichment analysis (GSEA) was performed using fold change and the fgsea R package with hallmark gene sets from the Molecular Signatures Database (31), which were downloaded using the msigdbr R package (https://CRAN.R-project.org/package=msigdbr). Normalized enrichment score was plotted based on GSEA and Benjamini–Hochberg adjusted *P* value. The subset of downregulation gene expression heatmap on the hallmark epithelial–mesenchymal transition (EMT) pathway was displayed. Raw and processed RNA-seq data were deposited in GEO (GSE253239).

### Lentivirus transduction

The *TDO2* ORF overexpression (OE) construct (Cat. # OHS6085-213574308, Horizon Discovery) and empty vector control PLX-304 (RRID: Addgene_25890) were obtained from the CCSB-Broad Lentiviral Expression Library (Horizon Discovery) at the University of Colorado Functional Genomics Facility. *TDO2*-KD shRNA constructs and shSCR control were obtained from MISSION(R) TRC Lentiviral shRNA Collection (MilliporeSigma), and sequences are listed in Supplementary Table S1. The lentiviral particles were generated by the UCCC Functional Genomics Shared Resource (RRID: SCR_021987). For virus transduction, SUM159PT or BT549 cells were seeded in a six-well plate and transduced with 500 µL to 1 mL of viral particles and then selected with 10 µg/mL blasticidin (Cat. # ant-bl, InvivoGen) or 10 μg/mL puromycin for 7 to 10 days. The TDO2 expression level after OE/KD was confirmed by immunoblotting or qPCR.

### Stable isotope tracing of TRP catabolism

^13^C_11_ L-TRP (CLM-4290-H-0.1) was purchased from Cambridge Isotope Laboratories, Inc. TDO2 inhibitor 680C91 (Cat. # SML-0287) and IDO1 inhibitor epacadostat (Cat. # S7910) were purchased from Sigma-Aldrich and Selleckchem, respectively. TDO2/IDO1 dual inhibitor AT-0174 was obtained from Antido Therapeutics, and the molecular structure has been published in Wu and colleagues (32). For isotope tracing, MDA-MB-453 cells were seeded in media supplemented with 16 mg/L labeled TRP following the indicated treatment. A total of 5 × 10^5^ MDA-MB-453 cells or 3 × 10^5^ BT549 cells were seeded in six-well plates and treated with 1 or 10 µmol/L AT-0174 under attached or suspension culture conditions. The cell pellets and corresponding conditioned media were harvested at 24 or 48 hours of treatment, cells counted, and samples snap-frozen. Metabolomic analyses were performed at the University of Colorado School of Medicine Mass Spectrometry Metabolomics Shared Resource Facility (RRID: SCR_021988). Medium samples were thawed on ice and diluted 1:25, and cell pellets were extracted at 2 × 10^6^ cells/mL in ice-cold lysis solution (5:3:2 MeOH:ACN:H_2_O) as previously described (33, 34). Samples were vortexed vigorously at 4°C for 30 minutes and then centrifuged at 4°C, 10,000 *g* for 10 minutes to isolate solids (35). The supernatants were transferred to autosampler vials and analyzed on a Vanquish UHPLC system coupled to a Q Exactive mass spectrometer (Thermo Fisher Scientific). Extracts were resolved over a Kinetex C18 column (150 × 2.1 mm × 1.7 µm; Phenomenex) at a flow rate of 450 µL/minute using 5-minute gradients in positive and negative ion polarity modes as previously described (35, 36). Samples were randomized and introduced to the mass spectrometry (MS) via electrospray ionization with the MS scanning in full MS mode (2 µscans) over the range of 65 to 950 *m*/*z* (10 µL injection for cells; 20 µL injection for media). Technical replicates were injected every 6 to 12 samples to ensure instrument stability (36). Metabolites were annotated and integrated using MAVEN (Princeton University; RRID: SCR_022491) in conjunction with the Kyoto Encyclopedia of Genes and Genomes database (RRID: SCR_012773). Peak quality was determined using blanks, technical mixes, and ^13^C_11_ natural abundance along with an in-house standard library (37). All studies were conducted in biological triplicate or above and unitless peak areas presented. The metabolomics data is publicly available (see data availability section).

### Transfection

BT549 sh*TDO2*-82 or BT549 sh*AhR*-85 cells were transfected with 1.5 µg pcDNA3.0-ZEB1 ORF using Lipofectamine 2000 (Cat. # 11668019, Invitrogen) according to the manufacturer’s protocol. After 24 hours, cells were harvested for use in invasion assays.

### Transwell invasion assay

For the assay, 24-well format 8.0-μm culture inserts (Cat. #353097, Falcon) were precoated with 200 µg/mL Cultrex UltiMatrix Reduced Growth Factor Basement Membrane Extract (Cat. # BME001-05, Bio-Techne). For BT549 shTDO2 or MDA-MB-453 shTDO2, cells were cultured under attached conditions. For BT549 or SUM159PT, cells were cultured under attached or suspension conditions and pretreated with 10 µmol/L AT-0174, 10 µmol/L AhR inhibitor StemRegenin 1 (Cat. # S2858, Selleckchem), or their combination for 48 hours. The next day, 5 × 10^4^ cells were seeded on the top of the inserts, and the lower compartment was supplemented with the complete media. After 24 hours, the inserts were fixed with 10% neutral buffered formalin and stained with 0.1% crystal violet, and the upper cells were removed by cotton swabs. For quantification, crystal violet–stained invaded cells were dissolved in 10% acetic acid/water and the absorbance measured at 570 nm.

### Anchorage-independent cell proliferation

BT549 or MDA-MB-453 sh*TDO2*/shSCR cells (3,000 cells/well) were seeded in 96-well ultralow attachment plates followed by AT-0174 treatment, and the cell growth was measured using CellTiter-Glo (Cat. # G7571, Promega), according to the manufacturer’s instructions at day 7.

### Immunoblotting and IHC

Immunoblotting: Cells were lysed using T-PER Tissue Protein Extraction Reagent (Cat. # 78510, Thermo Fisher Scientific) containing 1× Halt Protease Inhibitor Cocktail (Cat. # 87786, Thermo Fisher Scientific), and the protein concentration was quantified using the Pierce BCA Protein Assay Kit (Cat. # 23225, Thermo Fisher Scientific). Generally, 10 to 40 µg of total protein lysate was mixed with SDS loading buffer and resolved in SDS-PAGE and then transferred on Immobilon-FL membranes (Cat. # IPFL00010, MilliporeSigma). The membranes were then blocked with 3% Bovine Serum Albumin (BSA)/Tris-buffered saline with 0.1% Tween 20 (TBST) for 1 hour and hybridized with primary antibodies overnight at 4°C. After washing with TBST, the membranes were incubated with the secondary antibodies for 1 hour, and the fluorescent signal was detected using a LI-COR Odyssey Infrared Imaging System (RRID: SCR_013715). Primary antibodies used in this study were TDO2 (1:1,000 dilution, Cat. # ab259359, Abcam), IDO1 (1:1,000 dilution, Cat. # 86630S, Cell Signaling Technology), total NF-κB p65 (1:1,000 dilution, Cat. # 8242S, Cell Signaling Technology), phospho–NF-κB p65 (1:1,000 dilution, Cat. # 3033S, Cell Signaling Technology), ZEB1 (1:1,000 dilution, Cat. # 70512, Cell Signaling Technology), and α-tubulin (1:5,000 dilution, Cat. # T5168, Sigma-Aldrich; RRID: AB_477579). The secondary antibodies included Goat anti-Rabbit IgG Alexa Flour 680 (1:5,000 dilution, Cat. #21109, Thermo Fisher Scientific; RRID: AB_2535758) or IRDye 680RD Goat anti-Mouse IgG (1:5,000 dilution, Cat. # 926-68070, LI-COR Biosciences; RRID: AB_10956588). The protein densitometry was quantified using ImageJ software (RRID: SCR_003070). For IHC, cell pellets were paraffin embedded and subjected to IHC for TDO2 (1:50 dilution, Cat. # MABN1537, MilliporeSigma) as previously described (8).

### qRT-PCR

Total RNA was isolated using RNeasy Mini Kit (Cat. # 74104, QIAGEN). A measure of 1 µg of RNA was transcribed to cDNA using qScript cDNA SuperMix (Cat. # 95048, Quantabio) according to the manufacturer’s instructions. For qPCR, 10 µL of qPCR mixture contained 1.25 µL of cDNA (three times dilution from the reverse transcription cDNA product with nuclease-free water), 0.8 µL each of 10 µmol/L forward and reverse primers, 5 µL of PowerUp SYBR Green Master Mix (Cat. # 25742, Applied Biosystems/Thermo Fisher Scientific), and 2.15 µL nuclease-free water, and the SYBR amplification signal was detected using the 7500 Fast Real-Time PCR System (Applied Biosystems) and software (RRID: SCR_014596). GAPDH served as an internal control. Fold change was calculated based on 2^−ΔΔCT^ method using the formula ΔΔCt = [Ct (target) group − Ct (GAPDH) group] − [Ct (target) control − Ct (GAPDH) control], in which Ct represents threshold response and control signifies nontreatment or vehicle. The primers (5′–3′) were synthesized by Integrated DNA Technologies, and the sequences are listed in Supplementary Table S2.

### Public dataset analysis

The breast invasive carcinoma (The Cancer Genome Atlas Pan-Cancer Atlas; *n* = 1,084) dataset was analyzed using the cBioPortal platform (https://www.cbioportal.org/) to inquire the correlation of *AhR* and *ZEB1* mRNA expression. The Gene expression-based Outcome for Breast cancer Online platform (https://co.bmc.lu.se/gobo/gobo.pl) was utilized to analyze the expression of *AhR* and *ZEB1* in 51 breast cancer cell lines and the expression of *TDO2*, *AhR*, and *ZEB1* in 1,881 clinical breast tumors of different subtypes. Expression of multiple genes is averaged with median-centered analysis. The public dataset for AhR chromatin immunoprecipitation analysis (GSE127649, doi: 10.17989/ENCSR412ZDC) was used with inquiry of *ZEB1* and its upstream regulatory region.

### Statistical analysis

All the experiments in this study were performed in biological triplicate at three independent times and analyzed using GraphPad Prism v.9.4.1 (RRID: SCR_002798). A *P* value < 0.05 was set for statistical significance. All the statistical methods are described in the corresponding figure legends and presented with mean ± SD or SEM.

### Data availability

scRNA-seq data for MDA-MB-453 cells treated with vehicle or IL1β + TNFα were deposited in GEO as GSE237918. Bulk RNA-seq data for BT549 cells in suspension culture treated with AT-0174 or *TDO2* KD (sh*TDO2*) was deposited in GEO as GSE253239. The metabolomics data presented in this study is available at the NIH Common Fund's National Metabolomics Data Repository (NMDR) website, the Metabolomics Workbench (https://www.metabolomicsworkbench.org), where it has been assigned the following Study IDs: ST003484, ST003485, ST003486, ST003487, ST003488, ST003489, ST003490, ST003491.

## Results

### scRNA-seq demonstrates that inflammatory cytokines drive TRP catabolism through TDO2 upregulation

**Figure 1:**
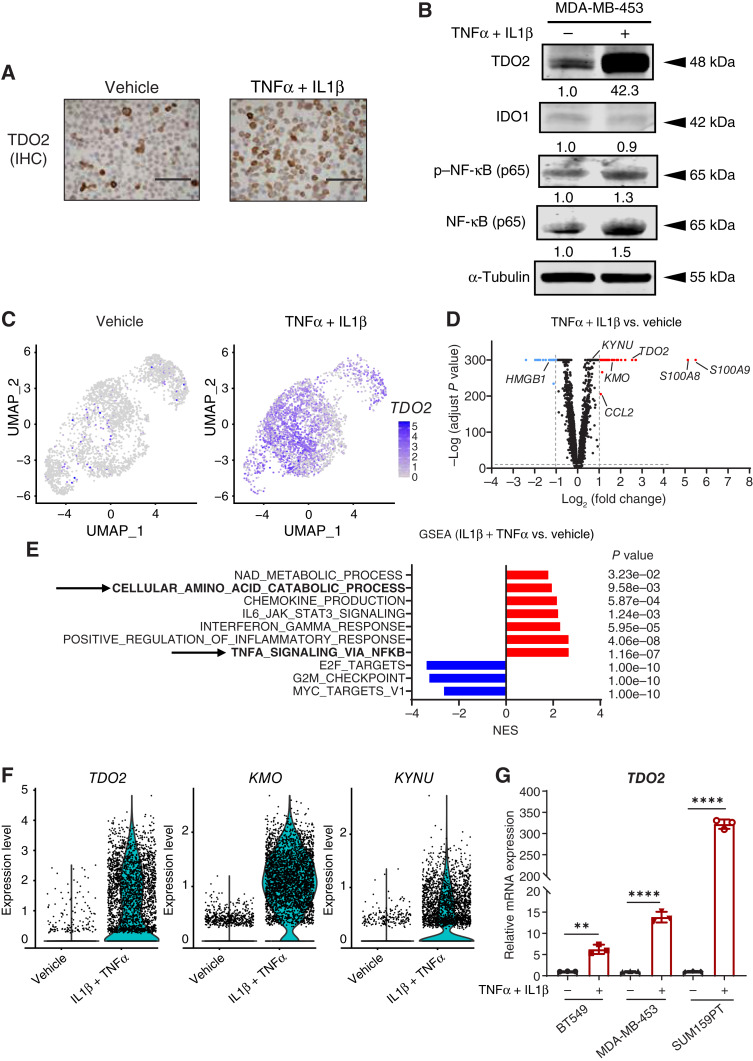
Inflammatory cytokines induce heterogeneous TDO2 expression in TNBC cells. **A,** IHC of TDO2 protein in MDA-MB-453 cells treated with vehicle or 10 ng/mL IL1β + TNFα for 48 hours. **B,** TDO2 protein in MDA-MB-453 treated with IL1β + TNFα or vehicle control for 48 hours and detected by immunoblotting. **C,** t-SNE graph showing *TDO2* in scRNA-seq on MDA-MB-453 cells treated with vehicle or IL1β + TNFα for 48 hours. **D,** Volcano plot showing gene fold change following vehicle or IL1β + TNFα treatment (*P* < 0.05; FDR < 0.05) with twofold or greater up- (red) or downregulated (blue) gene sets. **E,** Significant upregulated/downregulated genes identified using GSEA. **F,** scRNA-seq expression of TRP catabolism–associated genes *TDO2*, *KMO*, and *KYNU* in MDA-MB-453 treated with vehicle or IL1β + TNFα. **G,***TDO2* measured by qRT-PCR in three TNBC cell lines in response to vehicle or IL1β + TNFα treatment. Data are displayed as mean ± SD by two-way ANOVA analysis. *, *P* < 0.05; **, *P* < 0.01; ***, *P* < 0.001; ****, *P* < 0.0001. *KMO*, *kynurenine 3*-*monooxygenase*; NES, normalized enrichment score; t-SNE, t-distributed stochastic neighbor embedding; UMAP, Uniform Manifold Approximation and Projection.

We previously reported increased NF-κB activation in TNBC cells under anchorage-independent culture conditions compared with 2D culture conditions (5). TNBC cells surviving under anchorage-independent conditions increase TRP catabolism by specifically upregulating TDO2. Inflammatory cytokines IL1β and TNFα that activate NF-κB also increase TDO2 (38). As we had previously observed that even in TNBC cell lines, not all cells upregulate TDO2 in suspension culture with or without NF-κB–activating cytokines, we sought to identify the characteristics of cells that express and upregulate this enzyme. We utilized the MDA-MB-453 cell line, which has high endogenous TDO2 in approximately 20% of cells. The cells were treated with 10 ng/mL of IL1β and TNFα for 24 hours, and IHC for TDO2 was performed followed by scRNA-seq. Genes coexpressed in cells with *TDO2*-high expression at baseline without cytokine treatment are listed in Supplementary Table S10. An increase in TDO2 protein with inflammatory cytokines was confirmed by IHC and immunoblotting (Fig. 1A and B). Interestingly, the cytokine treatment upregulated *TDO2* mRNA and protein but did not upregulate *IDO1* (Fig. 1B), and these results were confirmed by Uniform Manifold Approximation and Projection analysis (Fig. 1C). Differential gene expression between IL1β + TNFα–treated and vehicle-treated cells demonstrated significant upregulation of genes associated with inflammatory cytokines, including *S100 calcium*-*binding protein A8*/*A9* and *chemokine ligand 2* along with the TRP catabolism enzymes *TDO2*, *kynureninase* (*KYNU*), and *kynurenine 3*-*monooxygenase* (Fig. 1D). The complete list of differentially expressed genes (with and without the addition of inflammatory cytokines) is provided in Supplementary Table S3. GSEA pathway analysis of scRNA-seq data in response to cytokine treatment and bulk RNA-seq data of MDA-MB-453 cells surviving in forced suspension culture both showed positive enrichment of TNFα signaling via NF-κB activation and cellular amino acid catabolic process (Fig. 1E; Supplementary Fig. S1). scRNA-seq also demonstrated increased *TDO2*, *KYNU*, and *kynurenine 3*-*monooxygenase* expression upon exposure to inflammatory cytokines (Fig. 1F). The significant upregulation of *TDO2* with NF-κB–activating cytokine treatment was also observed in the TNBC lines SUM159PT and BT549 (Fig. 1G).

### Cytokine treatment or anchorage-independent culture of TNBC cells increases TRP flux through the KYN pathway

**Figure 2:**
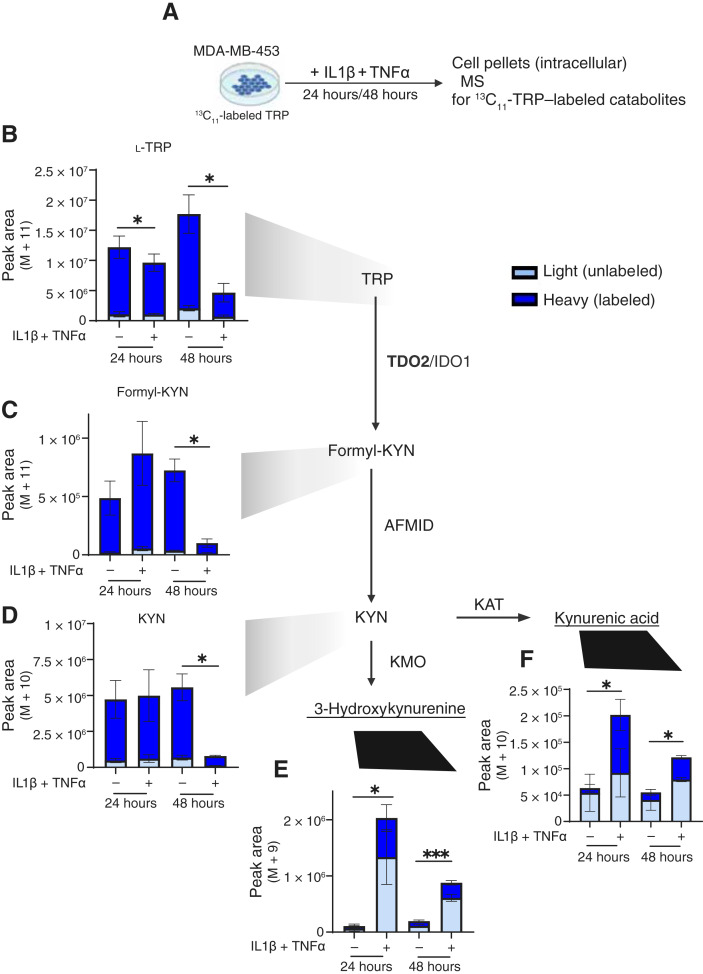
Tracing of TRP demonstrates that inflammatory cytokines enhance TRP catabolism and accumulation of KYN and other downstream catabolites. **A,** MDA-MB-453 cultured in TRP-depleted media with ^13^C_11_-labeled TRP for 8 hours were treated with vehicle or 10 ng/mL IL1β + TNFα for 24 or 48 hours, and cell pellets were lysed and subjected to MS. Labeled intracellular TRP catabolites (heavy, dark blue) or unlabeled (light, light blue) were measured. including (**B**) TRP, (**C**) formyl-KYN, (**D**) KYN, (**E**) 3-hydroxykynurenine, and (**F**) kynurenic acid shown as peak area. Biological replicates were conducted in each group, and data are displayed as mean ± SEM by Student *t* test analysis. *, *P* < 0.05; **, *P* < 0.01; ***, *P* < 0.001; ****, *P* < 0.0001.

To measure the flux of TRP through the KYN pathway over time, we performed tracing of labeled ^13^C_11_-TRP via MS in MDA-MB-453 cells cultured for 24 and 48 hours in TRP-depleted media supplemented with ^13^C_11_-TRP treated with or without IL1β + TNFα (Fig. 2A). Incorporation of heavy (^13^C_11_-labeled) TRP versus light (unlabeled) isotopologues of intracellular TRP (to test uptake) and downstream metabolites formyl-KYN and KYN (to test TRP catabolism) demonstrated a significant and rapid decrease in the level of TRP (Fig. 2B), formyl-KYN (Fig. 2C), and KYN (Fig. 2D) in response to IL1β + TNFα treatment as compared with vehicle-treated cells. The catabolites 3-hydroxykynurenine and kynurenic acid significantly increased with treatment (Fig. 2E and F). The percentage (%) isotope ^13^C_11_ labeled is shown in Supplementary Fig. S2. Tracing of labeled TRP showed that IL1β + TNFα increased flux through the TRP catabolism pathway, leading to a decrease in the levels of metabolites upstream of KYN and an accumulation of KYN and downstream metabolites. In addition, we performed ^13^C_11_-TRP tracing in MDA-MB-453 cells cultured under forced suspension conditions as compared with 2D culture (attached). Notably, intracellular TRP, formyl-KYN, and KYN increased in cells surviving in suspension as compared with the attached condition (Supplementary Fig. S3). Thus, both inflammatory cytokine treatment and anchorage independence increased the flux of TRP through the KYN pathway in TNBC, as a result of the increased TDO2.

### KD of *TDO2* can increase IDO1

**Figure 3:**
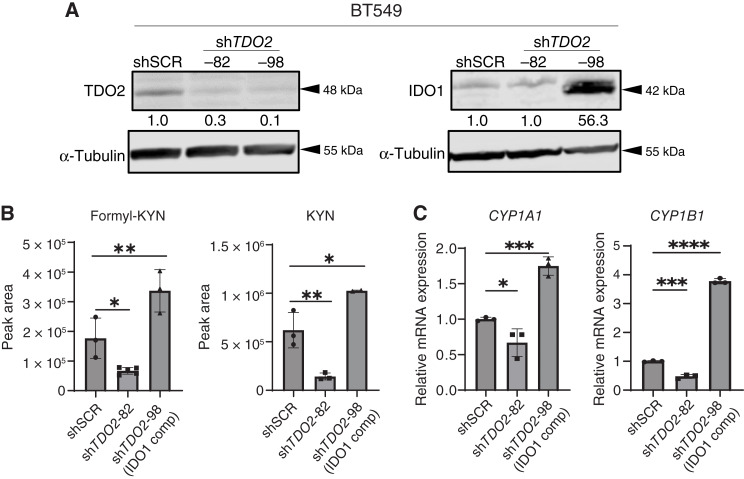
KD of *TDO2* can increase IDO1 expression and activity in a compensatory manner. Stable genetic KD *TDO2* (sh*TDO2*) or scramble control (shSCR) in BT549 cells. **A,** Immunoblot of TDO2 and IDO1. **B,** Catabolite level shown in peak area (mean ± SEM) of formyl-KYN and KYN as measured by steady-state metabolomics in conditioned media from BT549 with sh*TDO2* or shSCR control. **C,** Activated AhR-regulated genes *CYP1A1* and *CYP1B1* with and without shTDO2. Data are displayed as mean ± SD with one-way ANOVA analysis. *, *P* < 0.05; **, *P* < 0.01; ***, *P* < 0.001; ****, *P* < 0.0001.

To examine targeting TDO2 in invasive TNBC, BT549 cells were transduced with lentivirus carrying shRNA specific for *TDO2*. Immunoblotting confirmed the reduction in TDO2 at the protein level (Fig. 3A). Formyl-KYN and KYN were significantly reduced in the cells generated with one of the shRNA constructs (*TDO2*-82) but not reduced in cells created with another construct (sh*TDO2*-98; Fig. 3B). To investigate this, we interrogated IDO1 protein levels and discovered that IDO1 was 56-fold upregulated in the sh*TDO2*-98 cells (Fig. 3A). The sh*TDO2*-98 BT549 cells were thereafter denoted as having IDO1 compensation (or IDO1 comp). KD of *TDO2* also caused a significant reduction in the AhR target gene cytochrome P450 1A1/1B1 (*CYP1A1* and *CYP1B1*) in sh*TDO2*-082 cells without an increase in IDO1, but these genes were not reduced in the sh*TDO2*-98 cells (IDO1 comp) in which IDO1 showed a compensatory increase (Fig. 3C).

### TDO2/IDO1 dual inhibitor significantly reduced intracellular TRP and secreted KYN

**Figure 4:**
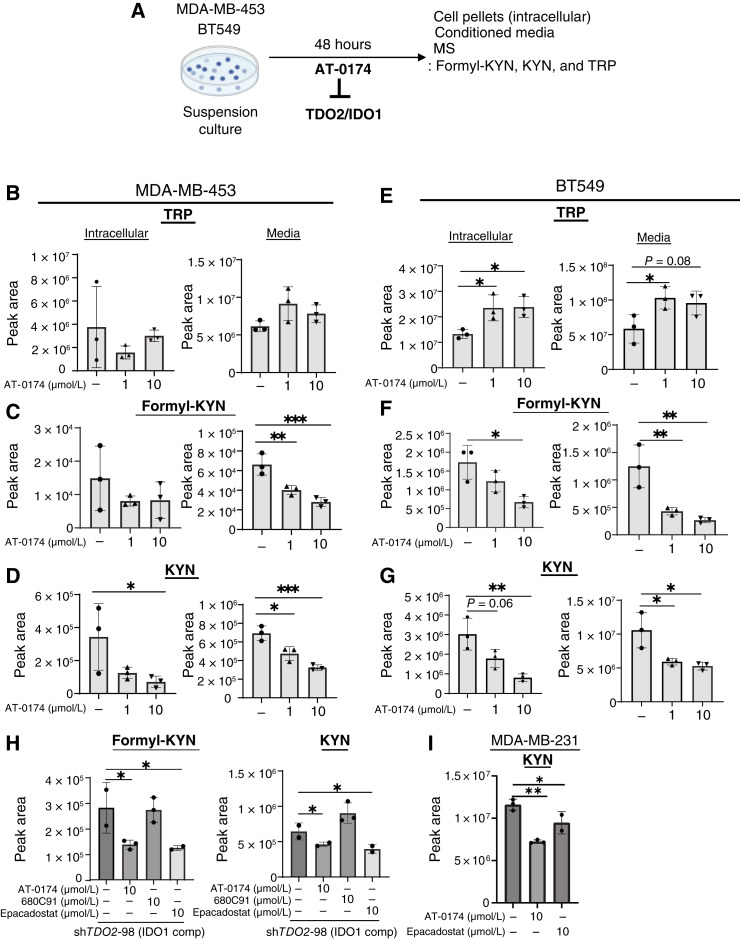
AT-0174, a dual TDO2/IDO1 inhibitor, decreases TRP catabolism in anchorage-independent TNBC cells expressing TDO2 or IDO1 and reduces both formyl-KYN or KYN. **A,** Schematic of experimental design. **B** and **E,** TRP; **C** and **F,** formyl-KYN; **D** and **G,** KYN levels in cells and conditioned media of MDA-MB-453 or BT549 following suspension culture with or without 1 µmol/L and 10 µmol/L of TDO2/IDO1 inhibitor AT-0174 treatment (**H**). BT549 sh*TDO2*-98 (IDO1 comp) was treated with 10 µmol/L AT-0174, 10 µmol/L TDO2 inhibitor 680C91, or 10 µmol/L IDO1 inhibitor epacadostat. **I,** KYN levels of MDA-MB-231 treated with vehicle (DMSO) control, 10 µmol/L AT-0174, or 10 µmol/L epacadostat. All treatments above were conducted for 48 hours, and conditioned media were analyzed for TRP catabolites and shown as peak area. Data are displayed as mean ± SEM by one-way ANOVA analysis. *, *P* < 0.05; **, *P* < 0.01; ***, *P* < 0.001; ****, *P* < 0.0001.

**Figure 5:**
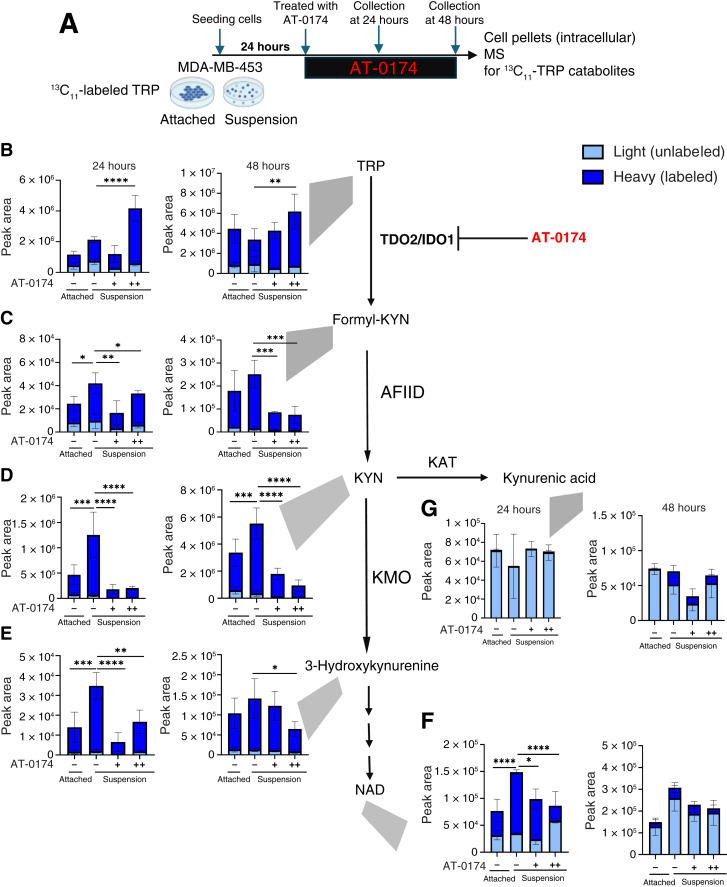
Tracing of TRP demonstrates that AT-0174, a dual TDO2/IDO1 inhibitor, decreases TRP catabolism. **A,** Schematic of the study design: cells in attached vs. forced suspension culture were incubated in ^13^C_11_-labeled TRP for 24 hours, and then suspended cells were treated with vehicle control (DMSO) or 1 µmol/L (“+”) or 10 µmol/L AT-0174 (“++”) for 24 or 48 hours at which time cells were harvested, lysed, and analyzed by MS. Intracellular heavy (labeled) TRP catabolites (dark blue) and light (unlabeled) TRP catabolites including (**B**) TRP, (**C**) formyl-KYN, (**D**) KYN, (**E**) 3-hydroxykynurenine, (**G**) kynurenic acid, and (**F**) NAD were measured and are represented as peak area. Biological replicates (*n* = 3) were conducted for each group, and data are displayed as mean ± SEM with two-way ANOVA analysis. *, *P* < 0.05; **, *P* < 0.01; **, *P* < 0.001; ****, *P* < 0.0001.

As we observed that IDO1 compensation is possible following *TDO2* KD, we tested the dual TDO2/IDO1 inhibitor AT-0174 that acts as a competitive TRP mimetic that can bind both enzymes (32). MDA-MB-453 and BT549 TNBC cells cultured in suspension (in which TDO2 is increased) were treated with or without two doses of AT-0174 (1 and 10 µmol/L; Fig. 4A). Although no significant TRP changes were observed in MDA-MB-453 (Fig. 4B), TRP significantly accumulated in the cells and media in BT549 (Fig. 4E) upon treatment with AT-0174, indicating that TRP was not being catabolized. We measured both intracellular levels of TRP and its catabolites, as well as the depletion of TRP or secretion of catabolites into the media. We found that secreted formyl-KYN significantly decreased with AT-0174 treatment at 1 and 10 µmol/L in MDA-MB-453 (Fig. 4C), whereas 10 µmol/L AT-0174 decreased both intracellular and secreted formyl-KYN in BT549 cells (Fig. 4F). AT-0174 at 10 µmol/L treatment significantly decreased intracellular and secreted KYN in both cell lines (Fig. 4D and G). We also stably OE *TDO2* in SUM159PT cells (Supplementary Fig. S4A) and found significantly higher secreted formyl-KYN and KYN compared with those secreted by control empty vector containing cells (Supplementary Fig. S4B). Furthermore, AT-0174 treatment significantly reduced KYN levels secreted into the media of *TDO2* OE cells (Supplementary Fig. S4C). To test whether the inhibitors against TDO2/IDO1 reduced TRP catabolites in the BT549 sh*TDO2*-98 cells (IDO1 comp), we utilized 680C91 (a selective TDO2 inhibitor), epacadostat (a selective IDO1 inhibitor), and the dual inhibitor of both IDO1 and TDO2 AT-0174 and conducted metabolomics to measure formyl-KYN and KYN. Epacadostat and AT-0174 significantly decreased secreted formyl-KYN and KYN in the media from sh*TDO2*-98 (IDO1 comp) cells, but 680C91 (the TDO2 inhibitor) did not (Fig. 4H), suggesting the upregulation of TRP catabolism because IDO1 compensation can be reduced using the inhibitors that target IDO1. We also used MDA-MB-231, which expresses high endogenous IDO1, and found AT-0174 or epacadostat reduces KYN (Fig. 4I). To further test the effect of AT-0174 on flux through the TRP catabolism pathway, we performed ^13^C_11_-TRP tracing in MDA-MB-453 under attached versus suspension culture conditions with or without 1 or 10 µmol/L AT-0174 treatment (Fig. 5A; Supplementary Fig. S5A). A measure of 10 µmol/L AT-0174 significantly increased intracellular TRP (Fig. 5B), but no significant changes in secreted TRP (Supplementary Fig. S5B). In contrast, AT-0174 decreased intracellular and secreted formyl-KYN and KYN levels (Fig. 5C and D; Supplementary Fig. S5C and S5D). No significance of kynurenic acid (Fig. 5G; Supplementary Fig. S5F) was observed in response to the treatment, suggesting that KYN was mainly processed to the direct downstream pathway. Notably, AT-0174 significantly decreased the production of 3-hydroxykynurenine (Fig. 5E; Supplementary Fig. S5E) and nicotinamide adenine dinucleotide (Fig. 5F), the terminal metabolite in the pathway, showing that dual inhibition of TDO2/IDO1 effectively reduced the flux of TRP through the KYN pathway.

### Dual inhibition of TDO2/IDO1 reduces TNBC invasive capacity by blocking KYN production and consequent activation of AhR

**Figure 6:**
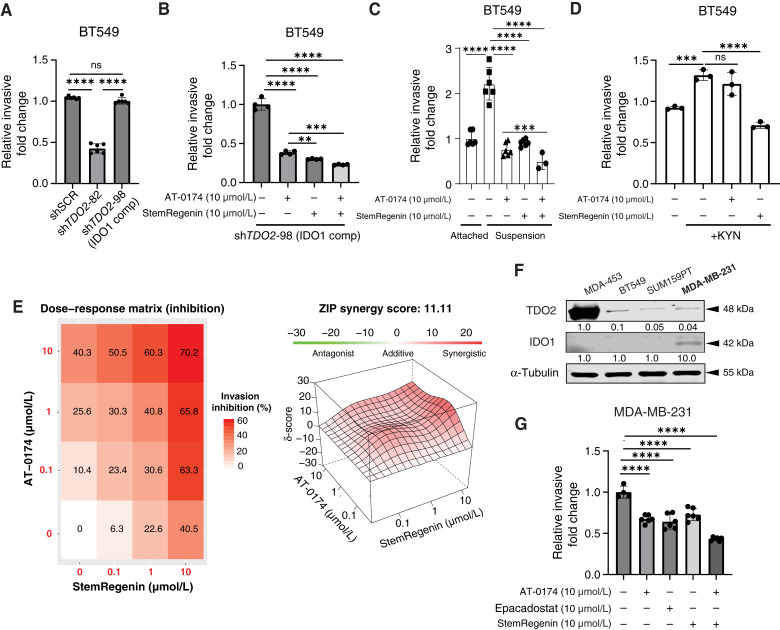
KD of *TDO2* reduces TNBC cell invasion except when IDO1 increases in a compensatory manner, and pharmacologic inhibition of TDO2/IDO1 reduces invasion and is synergistic with AhR inhibition. **A,** Invasion assay of scramble control (shSCR) or *TDO2*-KD (sh*TDO2*) BT549. **B,** Invasion assays following pretreatment and maintaining with 10 µmol/L AT-0174, 10 µmol/L AhR inhibitor StemRegenin, or combination on BT549 sh*TDO2*-98 (IDO1 comp) cells. **C,** BT549 cells under attached and suspension conditions were treated with 10 µmol/L of AT-0174 or StemRegenin. **D,** BT549 cells were treated with or without 10 µmol/L KYN in combination with 10 µmol/L AT-0174 and 10 µmol/L StemRegenin. **E,** Invasion inhibition (%) of BT549 cells treated with three doses (0.1, 1, and 10 µmol/L) of AT-0174, StemRegenin, or combination. Synergy score was calculated using SynergyFinder with the input of invasion inhibition. **F,** TDO2 and IDO1 protein expression in TNBC cell lines. **G,** MDA-MB-231 cells were treated with vehicle (DMSO) control, 10 µmol/L AT-0174, 10 µmol/L StemRegenin, 10 µmol/L epacadostat, or combination. All treatments were conducted for 48 hours, and the invasion through Cultrex was assayed by transwell for 24 hours. Invaded cells were stained with 0.5%/25% (v/v) crystal violet/methanol, dissolved in 10% acetic acid, and absorbance was measured. Data are displayed as mean ± SD with *t* test or one-way ANOVA analysis. *, *P* < 0.05; **, *P* < 0.01; ***, *P* < 0.001; ****, *P* < 0.0001.

To investigate whether dual inhibition of TDO2/IDO1 affects proliferation in anchorage-independent growth and invasion of TNBC, invasion assays were performed using transwell chambers coated with Cultrex, and invasive capacity was measured after 24 hours. *TDO2* KD (Supplementary Fig. S6A) or AT-0174 significantly reduced anchorage-independent growth and invasion in BT549 and MDA-MB-453 cell lines (Fig. 6A; Supplementary Fig. S6B and S6C). In contrast, the BT549 sh*TDO2*-98 (IDO1 comp) cells showed no significant difference in invasive capacity compared with shSCR control (Fig. 6A). Notably, treatment of BT549 sh*TDO2*-98 (IDO1 comp) cells with AT-0174 or the AhR inhibitor StemRegenin significantly reduced invasion, and the combination of these two inhibitors further reduced invasion (Fig. 6B). AT-0174 treatment also decreased invasion in BT549 and SUM159PT *TDO2* OE cells compared with vehicle control (Supplementary Fig. S7). To test invasive capability following anchorage-independent culture, when endogenous TDO2 increases, we seeded cells under forced suspension conditions for 24 hours and then treated with or without AT-0174, StemRegenin, or both. We found that AT-0174 or StemRegenin significantly reduced invasion (Fig. 6C; Supplementary Fig. S8A) and that treatment with both drugs further decreased invasion. Moreover, exogenous KYN significantly increased invasion, but treatment with AT-0174 did not affect invasion in the presence of KYN, as expected, because KYN binding to AhR is downstream of the action of AT-0174 on TDO2/IDO1 (Fig. 6D; Supplementary Fig. S8B). Conversely, the AhR inhibitor StemRegenin significantly reduced invasion in the context of KYN treatment as it inhibits AhR activity. To further investigate the effects of combinational treatment with the dual TDO2/IDO1 inhibitor and AhR inhibitor, we performed invasion assays with BT549 cells treated with three doses of AT-0174 and StemRegenin, alone or in combination, and found that the two drugs together resulted in synergistic effects with a synergy score of 11.11 (Fig. 6E). To elucidate if IDO1 also mediates invasion, we used MDA-MB-231 cells, which express high endogenous IDO1 (Fig. 6F), and treated them with AT-0174, epacadostat, StemRegenin, or the combination. We found that AT-0174 and epacadostat significantly also reduced invasion (Fig. 6G). Collectively, these data show that the inhibition of TDO2/IDO1 decreased invasion through KYN-mediated activation of AhR.

### Blocking TDO2/IDO1 reduces mesenchymal genes in TNBC lines

**Figure 7:**
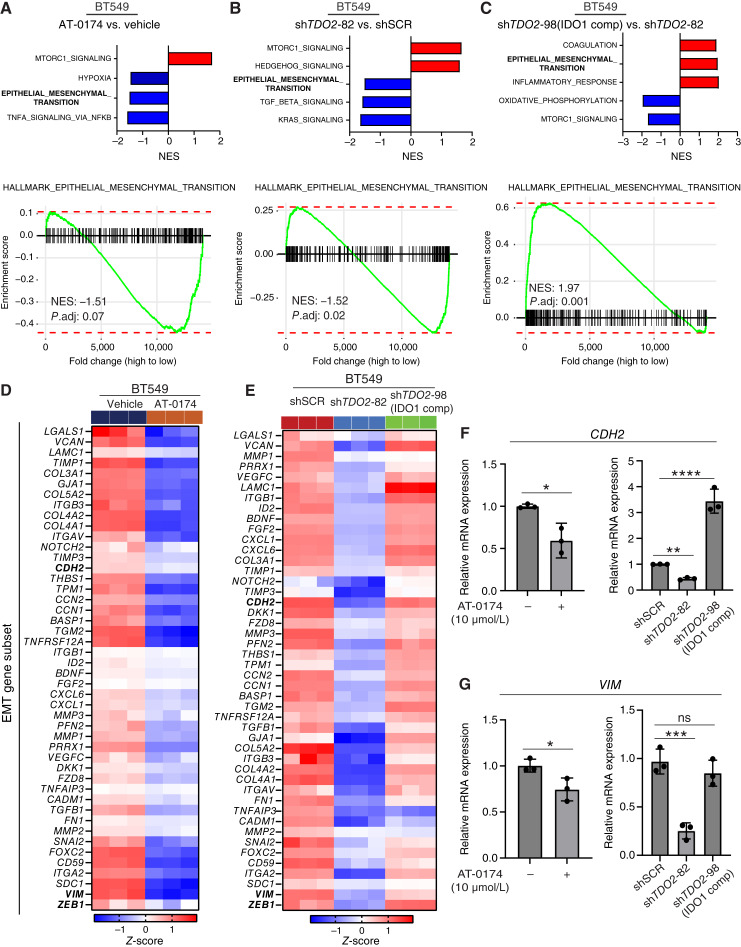
Pharmacologic inhibition of TDO2/IDO1 or *TDO2* KD reduces the EMT signature. **A–C,** GSEA was performed to analyze RNA-seq data from BT549 treated with AT-0174 or vehicle (AT-0174 vs. vehicle) for 48 hours, and BT549 with *TDO2* KD [sh*TDO2*-82 vs. shSCR and sh*TDO2*-98 (IDO1 comp) vs. sh*TDO2*-82], with biological triplicate for each condition. Significant pathways (*P* adj. < 0.05) with negative (blue) and positive (red) NES for the hallmark of EMT genes are shown. **D** and **E,** Heatmaps of the EMT gene subset from RNA-seq data with *z*-score-transformed CPM. **F** and **G,***VIM* and *CDH2* mRNA as by qPCR following AT-0174 treatment for 48 hours. Data are displayed as mean ± SD with *t* test or one-way ANOVA analysis. *, *P* < 0.05; **, *P* < 0.01; ***, *P* < 0.001; ****, *P* < 0.0001. *CDH2*, *N*-*cadherin*; CPM, counts per million; NES, normalized enrichment score; *VIM*, *vimentin*.

To elucidate the effect of TDO2 on TNBC invasive capacity, we performed RNA-seq in BT549 cells under anchorage-independent culture conditions with or without AT-0174 treatment and in BT549 cells with *TDO2* shRNA or control shRNA. Comprehensive differential gene expression and GSEA results are listed in Supplementary Tables S4–S9. GSEA showed a decrease in TNFα-mediated NF-κB signal gene signatures with AT-0174 treatment, suggesting that the TDO2/IDO1 inhibitor may reduce NF-κB activation during anchorage independence. In the sh*TDO2*-82 cells (with no IDO1 compensation), TGFβ and KRAS signatures are decreased compared with the control shRNA cells. Both sh*TDO2*-82 and pharmacologic inhibition with AT-0174 positively enriched the mTOR signature, a known response to TRP accumulation (39), indicating that TRP catabolism was inhibited. Importantly, the hallmark of the EMT pathway, particularly mesenchymal genes, was decreased with AT-0174 and sh*TDO2*-82 (Fig. 7A and B). Conversely, BT549 sh*TDO2*-98 (IDO1 comp) cells did not show a decrease in the mesenchymal gene signature or mTOR signal (Fig. 7C), suggesting that IDO1 activity may regulate EMT. Although somewhat controversial, the mesenchymal phenotype is generally associated with cancer invasive capacity (40–42). To further examine epithelial and mesenchymal gene sets, we examined the mesenchyme-associated genes *N*-*cadherin*, *vimentin*, and *ZEB1*. These genes were significantly decreased in response to AT-0174 treatment (Fig. 7D) as in sh*TDO2*-82 cells (Fig. 7E). However, in sh*TDO2*-98 (IDO1 comp), *N*-*cadherin* and *vimentin* did not decrease (Fig. 7F and G). RNA-seq also showed that AT-0174 treatment or *TDO2* KD decreased matrix metalloproteases *MMP1* and *MMP2* (Fig. 7D and E), and these proteases are known to be involved in invasion through extracellular matrix remodeling. Taken together, our RNA-seq data suggests that inhibition of TDO2 reduces mesenchymal gene sets, but mesenchymal genes were not reduced in cells that exhibit a compensatory increase in IDO1.

### Inhibition of TDO2 reduces invasion through ZEB1 in an AhR-dependent manner

**Figure 8:**
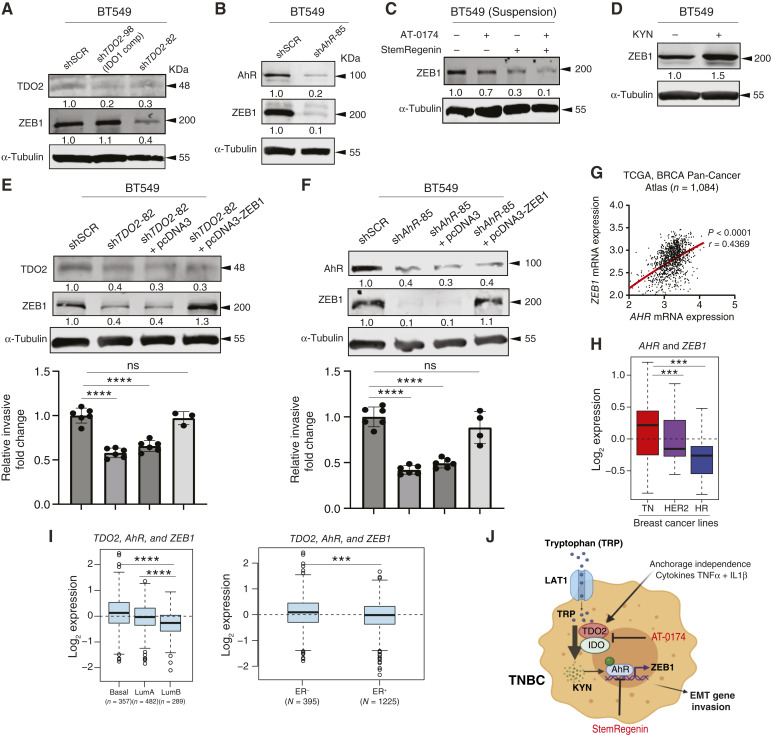
Inhibition TDO2 reduces invasion through AhR-mediated ZEB1 expression. ZEB1 protein expression in (**A**) BT549 with sh*TDO2* and (**B**) with sh*AhR* (**C**). BT549 cells cultured in suspension for 24 hours were treated with 10 µmol/L AT-0174 or 10 µmol/L StemRegenin for 48 hours. **D,** BT549 treated with 10 µmol/L KYN for 48 hours. **E** and **F,** Invasion assay for BT549 shSCR control and sh*TDO2*-82 or sh*AhR*-85 transfected with control pcDNA3.0 or pcDNA3.0-ZEB1 expression vector. **G,** Linear regression between *AhR a*nd *ZEB1* mRNA expression in TCGA Breast Cancer Atlas dataset with Pearson correlation analysis. **H,** Average *AhR* and Z*EB1* expression in TNBC and HER2-enriched and HR^+^ breast cancer cell lines (*n* = 51). **I,** Average expression of *TDO2*, *AhR*, and *ZEB1* from patients with basal-like and luminal A/B breast cancers. *TDO2*, *AhR*, and *ZEB1* average expression from patients stratified with ER^+/−^. The Gene expression-based Outcome for Breast cancer Online (GOBO) database and analyzed by one-way ANOVA or *t* test. *, *P* < 0.05; **, *P* < 0.01; ***, *P* < 0.001; ****, *P* < 0.0001. **J,** Schematic model showing TNBC with anchorage-independent conditions or inflammatory cytokine treatment can induce TDO2 and uptake TRP through LAT1, and dual inhibition of TDO2/IDO1 using AT-0174 or AhR inhibitor (StemRegenin) reduces invasion mediated by ZEB1. HR, hormone receptor; LAT1, L-type amino acid transporter 1; Lum, luminal; TCGA, The Cancer Genome Atlas.

Next, we explored the potential mechanism that inhibition of TDO2 decreases invasion. We focused on ZEB1 as we observed the decrease in ZEB1 expression in response to TDO2 inhibition by RNA-seq. ZEB1 drives the EMT by transcriptional suppression of the genes encoding the cell–cell adhesion molecule E-cadherin (43, 44), and it promotes invasion in many carcinomas (45–47). Importantly, the *ZEB1* promoter region contains predicted AhR-binding sites, and an AhR chromatin immunoprecipitation sequencing database (48) showed enrichment of AhR binding in the *ZEB1* transcript (Supplementary Fig. S9). Indeed, in the sh*TDO2*-82 BT549 cells, we found that both *ZEB1* mRNA and protein were significantly decreased but were not decreased in the sh*TDO2*-98 (IDO1 comp) cells (Fig. 8A; Supplementary Fig. S10A). IDO1 compensation restored *ZEB1* levels, possibly because of the increase in KYN (shown in Fig. 3B) and KYN-mediated AhR transcription of *ZEB1*. To elucidate if ZEB1 is driven by AhR, we knocked down AhR and found decreased ZEB1 (Fig. 8B). Also, AT-0174 and the AhR inhibitor StemRegenin 1, each significantly reduced ZEB1 mRNA and protein and the combination caused a further decrease in protein expression (Fig. 8C; Supplementary Fig. S10B). To address whether KYN-mediated AhR transcription drives ZEB1 expression, we used exogenous KYN and found that treatment with KYN increased ZEB1 in BT549 cells (Fig. 8D; Supplementary Fig. S10C), suggesting that KYN drives AhR transcription/translation to increase ZEB1 expression. To test if the TDO2/AhR signaling axis drives TNBC invasion through ZEB1, we transfected the ZEB1 overexpression vector in the TDO2-KD (sh*TDO2*) or AhR-KD (sh*AhR*) cells. ZEB1 OE restored the invasive capability under both conditions (Fig. 8E and F). These data demonstrate that TDO2/KYN/AhR drives TNBC invasion through ZEB1.

### *TDO2*, *AhR*, and *ZEB1* are expressed in TNBC clinical datasets

The Cancer Genome Atlas dataset (Pan-Cancer Atlas) from patients with breast cancer showed a significant positive correlation between *AhR* and *ZEB1* (Fig. 8G). Moreover, the Gene expression-based Outcome for Breast cancer Online dataset (49) showed that TNBC cell lines expressed a higher average of *AhR* and *ZEB1* expression compared with that in hormone receptor–positive HER2^+^ breast cancer cell lines (Fig. 8H). Furthermore, the average of the expression of *TDO2*, *AhR*, and *ZEB1* genes is higher in basal-like breast cancer (TNBC) than that in luminal A/B [estrogen receptor–positive (ER^+^)] breast cancer and when generally comparing ER^−^ versus ER^+^ tumors (Fig. 8I). The individual gene expression in the dataset above is shown in Supplementary Fig. S11A–S11C. Thus, the TDO2/AhR/ZEB1 axis may serve as a therapeutic target in TNBC. Although primary breast cancer tumors were examined in these clinical datasets for expression levels of *TDO2*, *AhR*, and *ZEB1*, these genes may be expressed at even higher levels in circulating tumor cells (anchorage-independent condition).

## Discussion

In our prior study (8), we found that anchorage-independent survival led to an increase in L-type amino acid transporter 1 and an increase in TDO2 (8). Here, we show that both anchorage independence and stimulation with inflammatory cytokines activate the NF-κB signaling cascade, leading to upregulation of TDO2 but not IDO1-mediated TRP catabolism. The IL6/STAT3 axis also increases TDO2, and consequent KYN production leads to the activation of AhR in response to inflammation in various types of cancer (11, 50). IHC and scRNA-seq solidified our previous observation that TNFα and IL1β increase *TDO2* mRNA and protein levels in a heterogeneous fashion, even within TNBC cell lines (8, 13).

Here, we find that IDO1 can be induced in the context of TDO2 suppression. In lung cancer cells, IDO1 KD led to an increase in TDO2 (32), suggesting that both enzymes can increase in a compensatory manner. Although the exact feedback mechanism underlying the ability of these two enzymes to sense and compensate for each other remains to be determined, these results emphasize the need for a dual inhibitor that targets both enzymes. Here, we tested one such inhibitor, AT-0174 (32).

Structurally, TDO2 and IDO1 are distinct but share similarities in the catalytic sites that process TRP (14, 51). Prior inhibitors tested in the clinic specifically targeted IDO1, to determine if inhibiting the production of KYN would enhance the activity of immune checkpoint blockade therapies such as anti–PD-L1/PD-1 (18, 52). The rationale for the combination therapy was due to secreted IFNγ from cytotoxic T cells driving expression of PD-L1/PD-1 and TDO2/IDO1 in cancer cells in an immunosuppressive feedback loop (53) because KYN binding to AhR inhibits the viability and function of cytotoxic T cells but expands regulatory T cells (54). However, phase III clinical trials (NCT02752074/ECHO-301/KEYNOTE-252) combining epacadostat (that specifically targets IDO) with pembrolizumab (anti–PD-1) in advanced melanoma resulted in no significant improvement in patient outcomes (55, 56). It is possible that the tumors in these epacadostat trials were more dependent on TDO2 than IDO1 or that TDO2 activity/dependency increased in the face of targeting IDO1, leading to an inefficient reduction of KYN production. Our recent study in ovarian cancer demonstrated that TDO2/IDO1 targeting alone attenuates PD-L1 expression (13). The TDO2 inhibitor 680C91 has been used in preclinical studies (14, 57) but is not practical *in vivo* given its poor pharmacokinetic properties. Here, the dual TDO2/IDO1 inhibitor AT-0174 blocks TRP catabolism in TNBC cells dependent on TDO2, in cells that acquire compensatory IDO1, and in the MDA-MB-231 cells that express more IDO1.

We previously reported that *TDO2* is targeted by microRNA-200c, which is lost in TNBC, permitting TDO2 expression (38). Significantly, the loss or silencing of miR-200c in TNBC facilitates EMT because members of the miRNA-200 family directly target and suppress *ZEB1* and many other mesenchymal and neuronal (non-epithelial) genes (58, 59). In this study, we find that both dual TDO2/IDO1 inhibition and inhibition of AhR significantly reduce ZEB1. Importantly, TDO2 KD or the dual TDO2/IDO1 inhibitor reduced TNBC invasive capacity by reducing levels of ZEB1 transcript and protein. In cells in which IDO1 increases in a compensatory manner (upon TDO2 KD), invasive capacity was restored, indicating that both enzymes mediate TNBC invasion, likely through KYN-mediated activation of AhR, as AhR inhibition also decreased invasion and exogenous KYN increased invasion.

In summary, here, we demonstrate that both inflammatory cytokines and anchorage-independent survival upregulate TDO2 expression and activity in TNBC cells and that TDO2 inhibition can lead to IDO1 compensation. Thus, dual inhibition of both enzymes is necessary to block TRP conversion to KYN in TNBC effectively. The TDO2/IDO1 dual inhibitor AT-0174, which can be orally administered and is in a phase I clinical trial (ACTRN12623000956606), blocked TRP catabolism and inhibited KYN/AhR-mediated TNBC invasive capacity. We have also tested this inhibitor in ovarian cancer preclinical models and observed that AT-0174 enhanced the antitumor effects of chemotherapy (13). As the present study was limited to in depth *in vitro* analyses of TRP catabolism via the KYN pathway in TNBC, we are currently designing *in vivo* studies to test the effects of dual TDO2/IDO1 inhibition on both primary TNBC and metastasis and ways to analyze direct effects versus effects on the immune system in immune-competent models. *In vivo* modeling will also determine if dual TDO2/IDO1 inhibition influences primary tumor growth or whether its utility as an adjuvant therapy will reduce the risk of recurrence as a metastatic disease.

## Supplementary Material

Supplementary Table S1 shRNA used in this study.

Supplementary Table S2 qPCR primers used in this study

Supplementary Table S3 Differential gene expressions of scRNA-seq for MDA-MB-453 treated with IL1β+TNFα vs. Vehicle

Supplementary Table S4 Significant GSEA of Bulk RNA-seq for BT549 (under suspension condition) treated with AT-0174 vs. Vehicle

Supplementary Table S5 Differential gene expression list of Bulk RNA-seq for BT549 (under suspension condition) treated with AT-0174 vs. Vehicle

Supplementary Table S6 Significant GSEA full list of Bulk RNA-seq for BT549 shTDO2-82 vs. shSCR control

Supplementary Table S7 Differential gene expression list of Bulk RNA-seq for BT549 shTDO2-82 vs. shSCR Control

Supplementary Table S8 Significant GSEA full list of Bulk RNA-seq for BT549 shTDO2-98 (IDO1 comp) vs. shTDO2-82

Supplementary Table S9 Differential gene expression list of Bulk RNA-seq for BT549 shTDO2-98 (IDO1 comp) vs. shTDO2-82

Supplementary Table S10 Gene co-expression with TDO2 list of single-cell RNA-seq for MDA-MB-453 treated with Vehicle

Supplementary Figure S1 Gene enrichment set analysis (GSEA) for MDA-MB-453 forced suspension culture vs. attached culture.

Supplementary Figure S2 Increased tryptophan catabolism and accumulation of kynurenine and other downstream catabolites.

Supplementary Figure S3 Tryptophan catabolism to kynurenine was enhanced in cells surviving in the anchorage independent condition over time.

Supplementary Figure S4 Compound AT-0174 decreased secreted KYN in TDO2 overexpression (OE) cells.

Supplementary Figure S5 AT-0174 significantly decreased extracellular tryptophan catabolites produced by MDA-MBA-453 cells.

Supplementary Figure S6 Pharmacologic inhibition or genetic knockdown of TDO2 reduces TNBC anchorage independent growth.

Supplementary Figure S7 AT-0174 reduced invasion in TDO2 OE cells.

Supplementary Figure S8 Treatment of AT-0174, epacadostat or StemRegenin reduces invasion in SUM159PT cells.

Supplementary Figure S9 The AhR ChIP seq dataset shows high enrichments at the promoter region of ZEB1 transcript.

Supplementary Figure S10 Genetic knockdown or pharmacological inhibition of TDO2 or AhR decreased ZEB1 mRNA expression but increased while IDO1 compensated; KYN treatment increased ZEB1 expression.

Supplementary Figure S11 AhR, ZEB1 and TDO2 expression in clinical breast cancer database.
