# Supplementary figures and images for: Blocking Tryptophan Catabolism Reduces Triple-Negative Breast Cancer Invasive Capacity
Source: Cancer Res Commun. 2024 Oct 16;4(10):2699–713. doi: 10.1158/2767-9764.CRC-24-0272 (PMC11484926; doi:10.1158/2767-9764.CRC-24-0272)

**
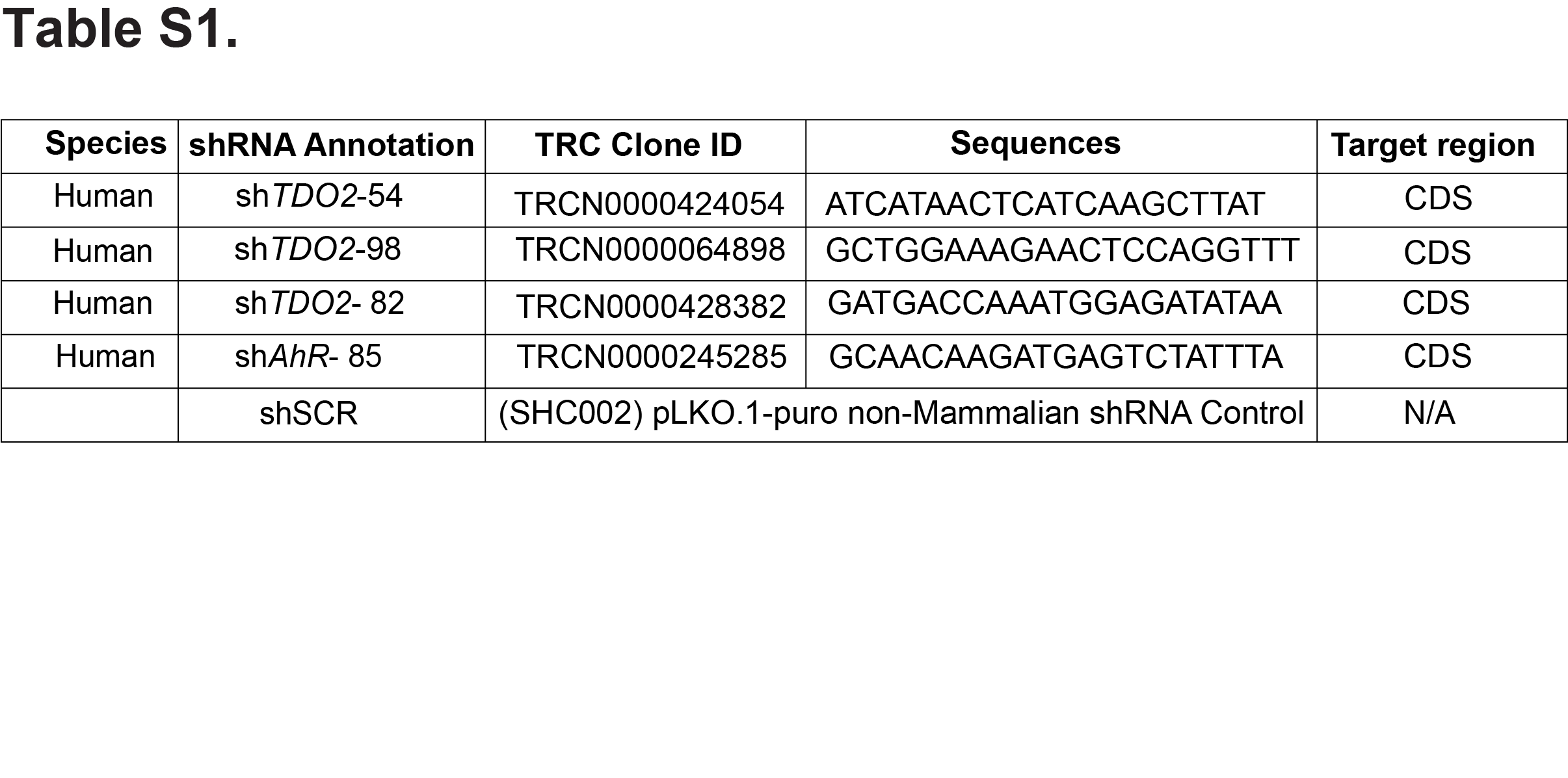
**

**Supplementary Table S1. shRNA used in this study.**

Supplement: Supplementary Table S1 — shRNA used in this study. [file crc-24-0272_supplementary_table_s1_suppst1.docx]
