# Supplementary material for: Blocking Tryptophan Catabolism Reduces Triple-Negative Breast Cancer Invasive Capacity
Source: Cancer Res Commun. 2024 Oct 16;4(10):2699–713. doi: 10.1158/2767-9764.CRC-24-0272 (PMC11484926; doi:10.1158/2767-9764.CRC-24-0272)
Supplement: Supplementary Table S2 — qPCR primers used in this study [file crc-24-0272_supplementary_table_s2_suppst2.docx]

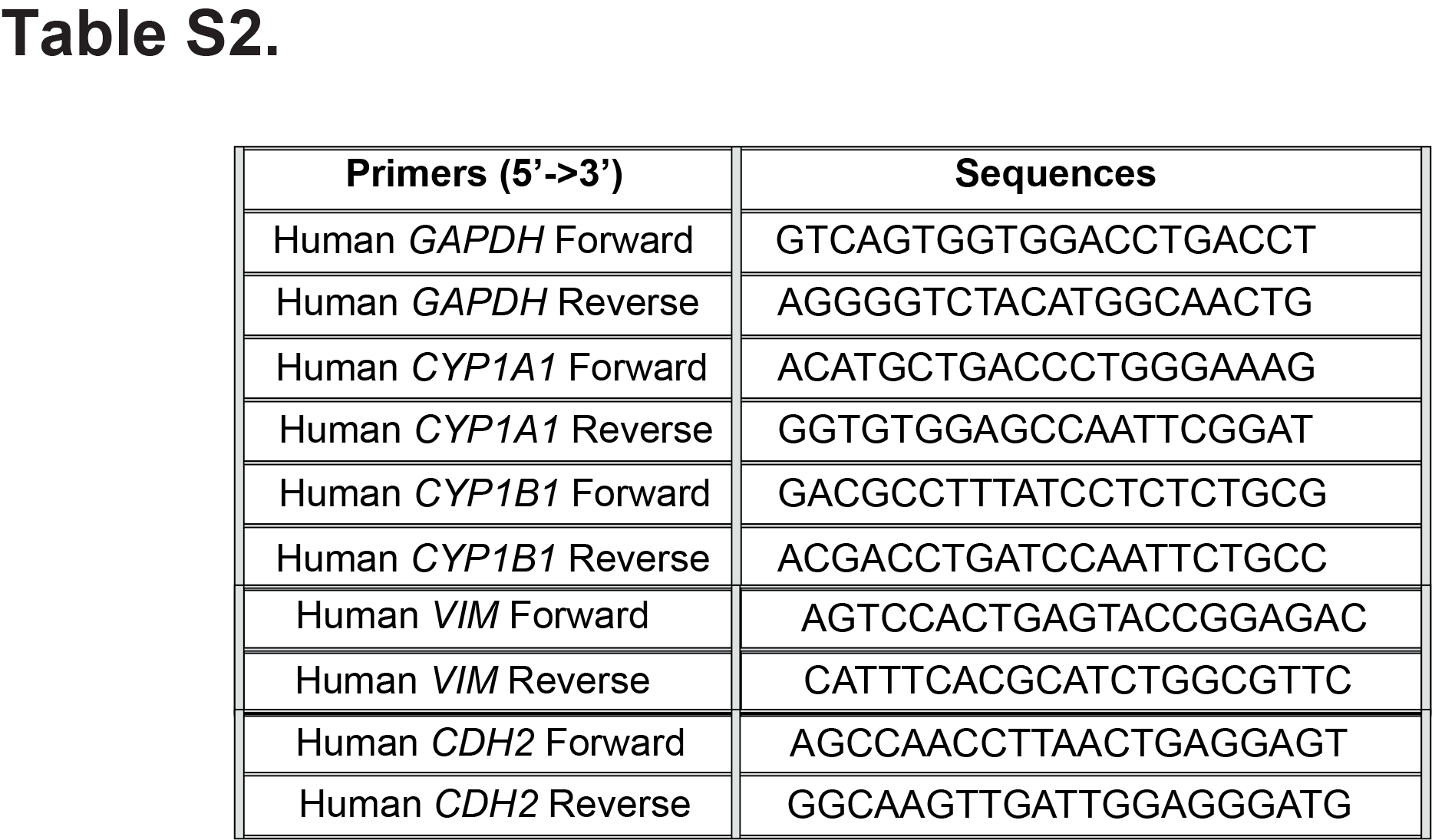


**Supplementary Table S2. qPCR primers used in this study.** The primers for corresponding genes were listed.
