# Supplementary material for: Blocking Tryptophan Catabolism Reduces Triple-Negative Breast Cancer Invasive Capacity
Source: Cancer Res Commun. 2024 Oct 16;4(10):2699–713. doi: 10.1158/2767-9764.CRC-24-0272 (PMC11484926; doi:10.1158/2767-9764.CRC-24-0272)
Supplement: Supplementary Figure S1 — Gene enrichment set analysis (GSEA) for MDA-MB-453 forced suspension culture vs. attached culture. [file crc-24-0272_supplementary_figure_s1_suppsf1.docx]

**Supplementary Figures and Tables**

**
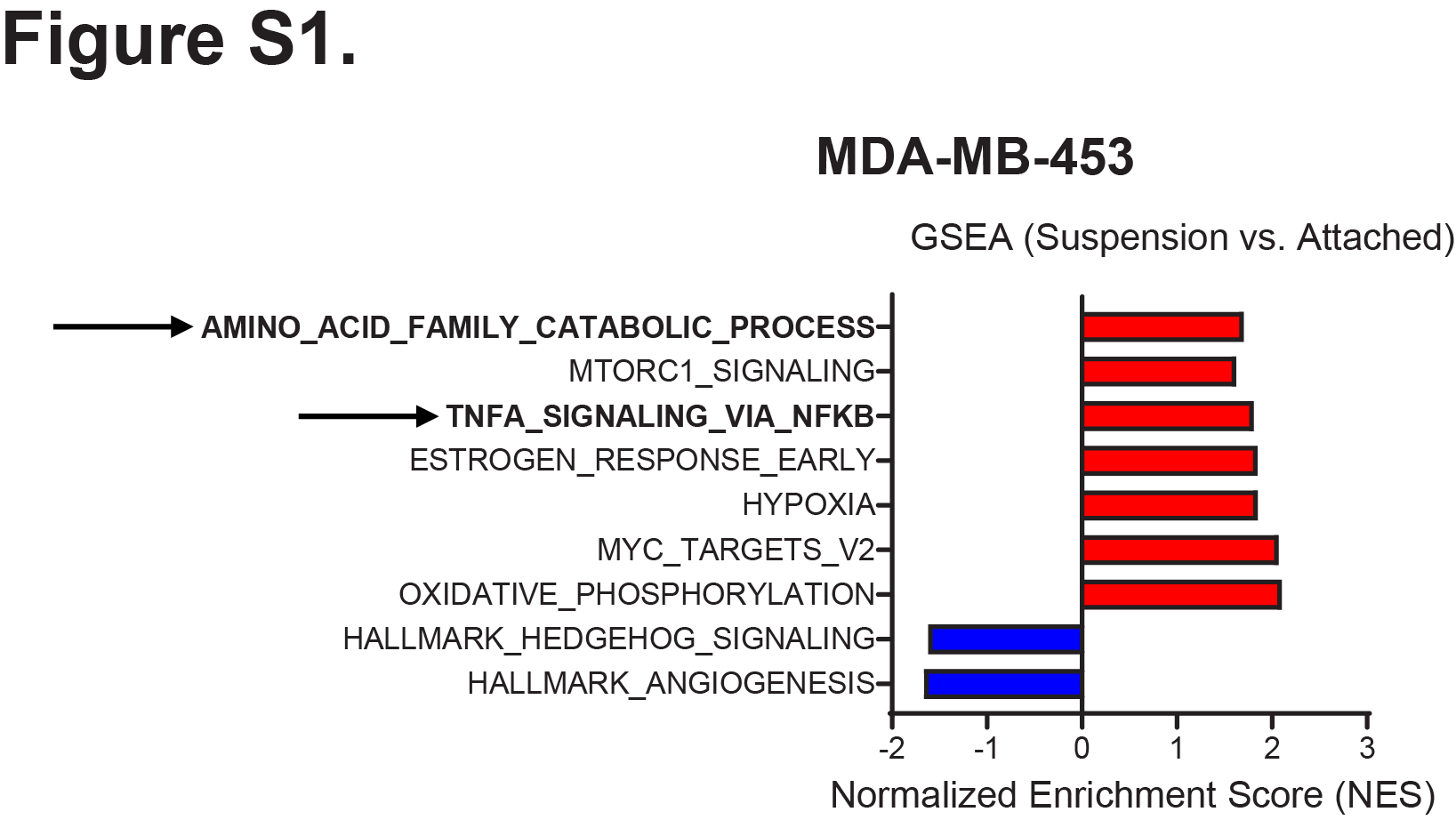
**

**Supplementary Figure S1. Gene enrichment set analysis (GSEA) for MDA-MB-453 forced suspension culture vs. attached culture.** MDA-MB-453 cells were cultured in suspension and regular attached conditions. RNA was collected at 48 hours and subjected to RNA-seq. The GSEA was performed, and top positive and negative enrichment pathways were listed in comparing forced suspension culture with attached culture.
