# Supplementary material for: Blocking Tryptophan Catabolism Reduces Triple-Negative Breast Cancer Invasive Capacity
Source: Cancer Res Commun. 2024 Oct 16;4(10):2699–713. doi: 10.1158/2767-9764.CRC-24-0272 (PMC11484926; doi:10.1158/2767-9764.CRC-24-0272)
Supplement: Supplementary Figure S2 — Increased tryptophan catabolism and accumulation of kynurenine and other downstream catabolites. [file crc-24-0272_supplementary_figure_s2_suppsf2.docx]

**
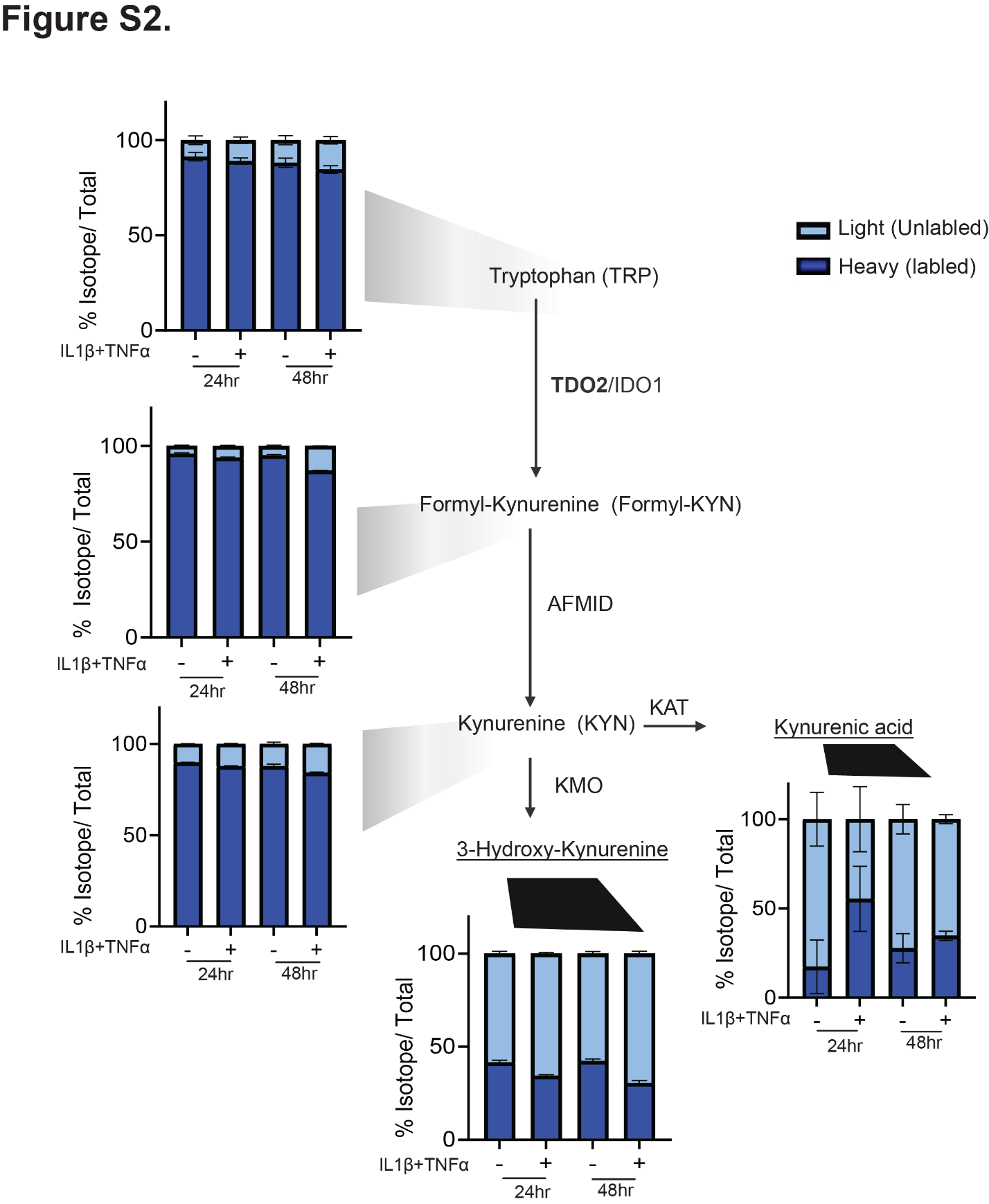
**

**Supplementary Figure S2. Increased tryptophan catabolism and accumulation of kynurenine and other downstream catabolites**. The data was identical to Figure 2 but presented with %^13^C_11_ labeled isotope/ Total (labeled+ unlabeled) corresponding metabolites. Labeled (heavy, dark blue) or unlabeled (light, light blue) intracellular metabolites: Tryptophan (TRP), Formyl-Kynurenine (Formyl-KYN), Kynurenine (KYN), 3-Hydro-Kynurenine and Kynurenic acid were shown in %. Biological replicates were conducted in each condition, and data displayed as Mean± SEM.
