# Supplementary material for: Blocking Tryptophan Catabolism Reduces Triple-Negative Breast Cancer Invasive Capacity
Source: Cancer Res Commun. 2024 Oct 16;4(10):2699–713. doi: 10.1158/2767-9764.CRC-24-0272 (PMC11484926; doi:10.1158/2767-9764.CRC-24-0272)
Supplement: Supplementary Figure S3 — Tryptophan catabolism to kynurenine was enhanced in cells surviving in the anchorage independent condition over time. [file crc-24-0272_supplementary_figure_s3_suppsf3.docx]

**
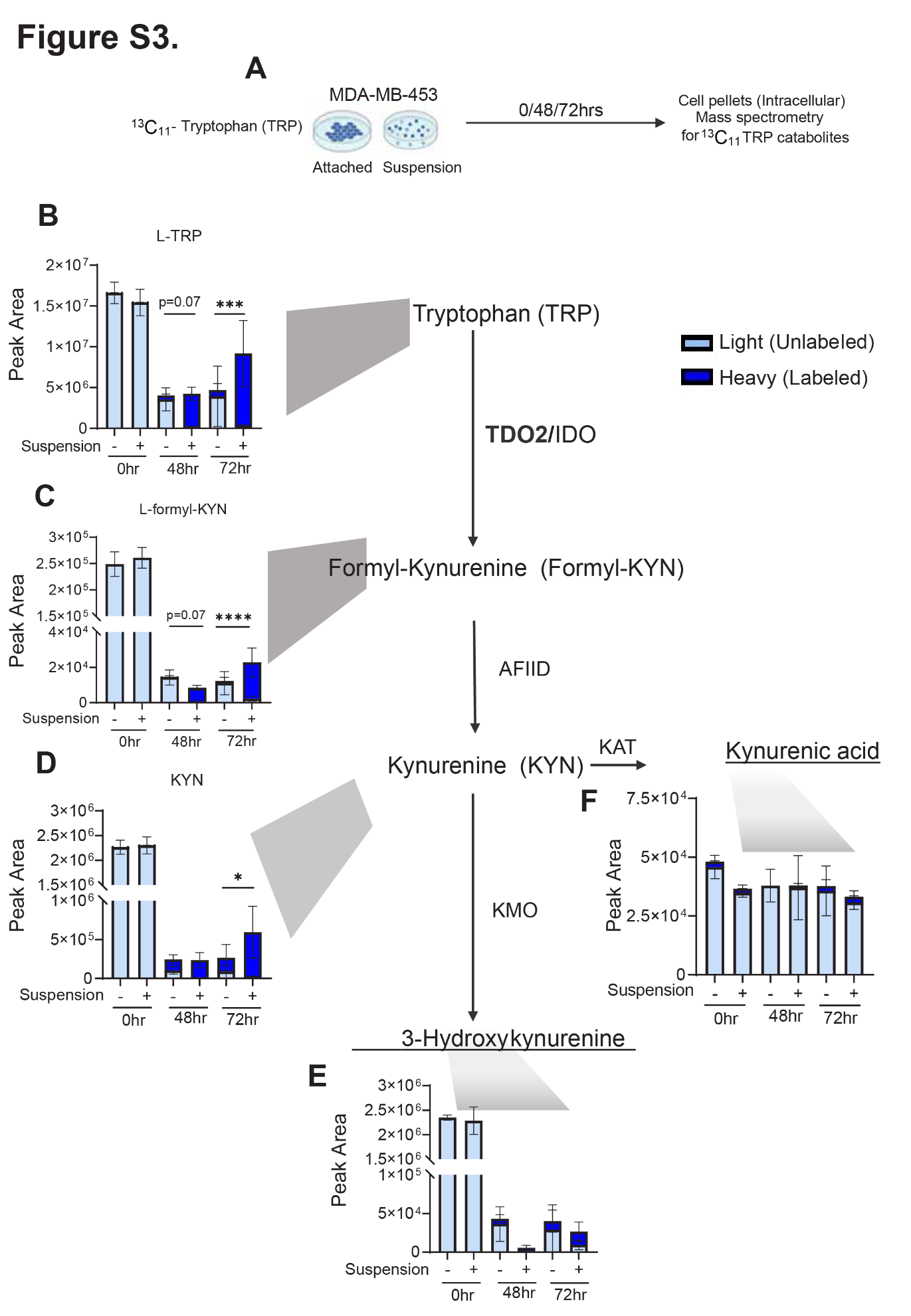
**

**Supplementary Figure S3.** **Tryptophan catabolism to kynurenine was enhanced in cells surviving in the anchorage independent condition over time.**  A. MDA-MB-453 were cultured in attached and suspension condition for 0hr, 48hrs or 72hrs, and cell pellets were subjected to mass spectrum to measure intracellular ^13^C_11_ labeled tryptophan catabolites. B, Tryptophan (TRP); C, Formyl-Kynurenine (Formyl-KYN); D, Kynurenine (KYN); E, 3-Hydro-Kynurenine; F, Kynurenic acid. The fractions were shown in the peak area. The biological replicates were conducted in each group, and the data displayed Mean± SEM with Two-way ANOVA analysis *: p<0.05, **p<0.01, ***p<0.001, ****p<0.0001.
