# Supplementary material for: Blocking Tryptophan Catabolism Reduces Triple-Negative Breast Cancer Invasive Capacity
Source: Cancer Res Commun. 2024 Oct 16;4(10):2699–713. doi: 10.1158/2767-9764.CRC-24-0272 (PMC11484926; doi:10.1158/2767-9764.CRC-24-0272)
Supplement: Supplementary Figure S4 — Compound AT-0174 decreased secreted KYN in TDO2 overexpression (OE) cells. [file crc-24-0272_supplementary_figure_s4_suppsf4.docx]

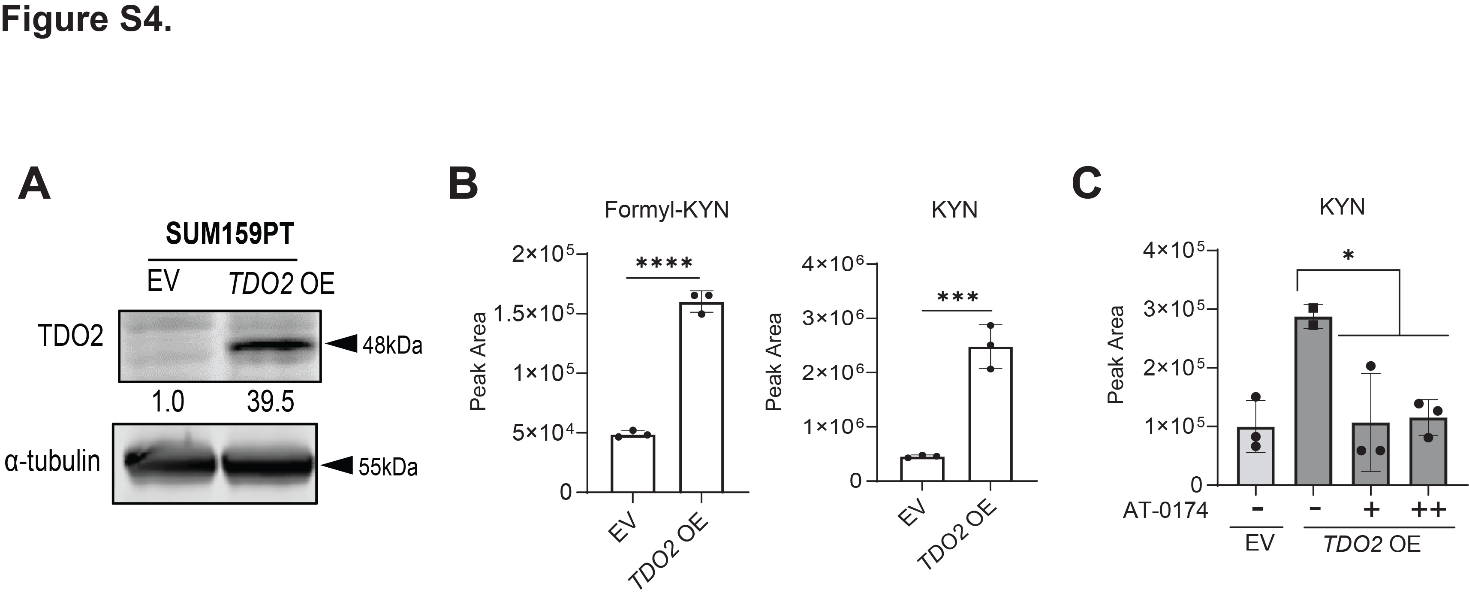


**Supplementary Figure S4. Compound AT-0174 decreased secreted KYN in *TDO2* overexpression (OE) cells.** A. immunoblot for SUM159PT *TDO2* OE and empty vector (EV) control cells. B. Mass spectrometry measurement of secreted formyl- kynurenine and kynurenine (KYN) for condition media from SUM159PT *TDO2* OE/EV. C. SUM159PT *TDO2* OE/EV treated with/ without 1μM (+), 10μM (++) of AT-0174 for 48 hours. Mean± SEM with t-test or One-way ANOVA analysis *: p<0.05, **p<0.01, ***p<0.001, ****p<0.0001.
