# Supplementary material for: Blocking Tryptophan Catabolism Reduces Triple-Negative Breast Cancer Invasive Capacity
Source: Cancer Res Commun. 2024 Oct 16;4(10):2699–713. doi: 10.1158/2767-9764.CRC-24-0272 (PMC11484926; doi:10.1158/2767-9764.CRC-24-0272)
Supplement: Supplementary Figure S5 — AT-0174 significantly decreased extracellular tryptophan catabolites produced by MDA-MBA-453 cells. [file crc-24-0272_supplementary_figure_s5_suppsf5.docx]

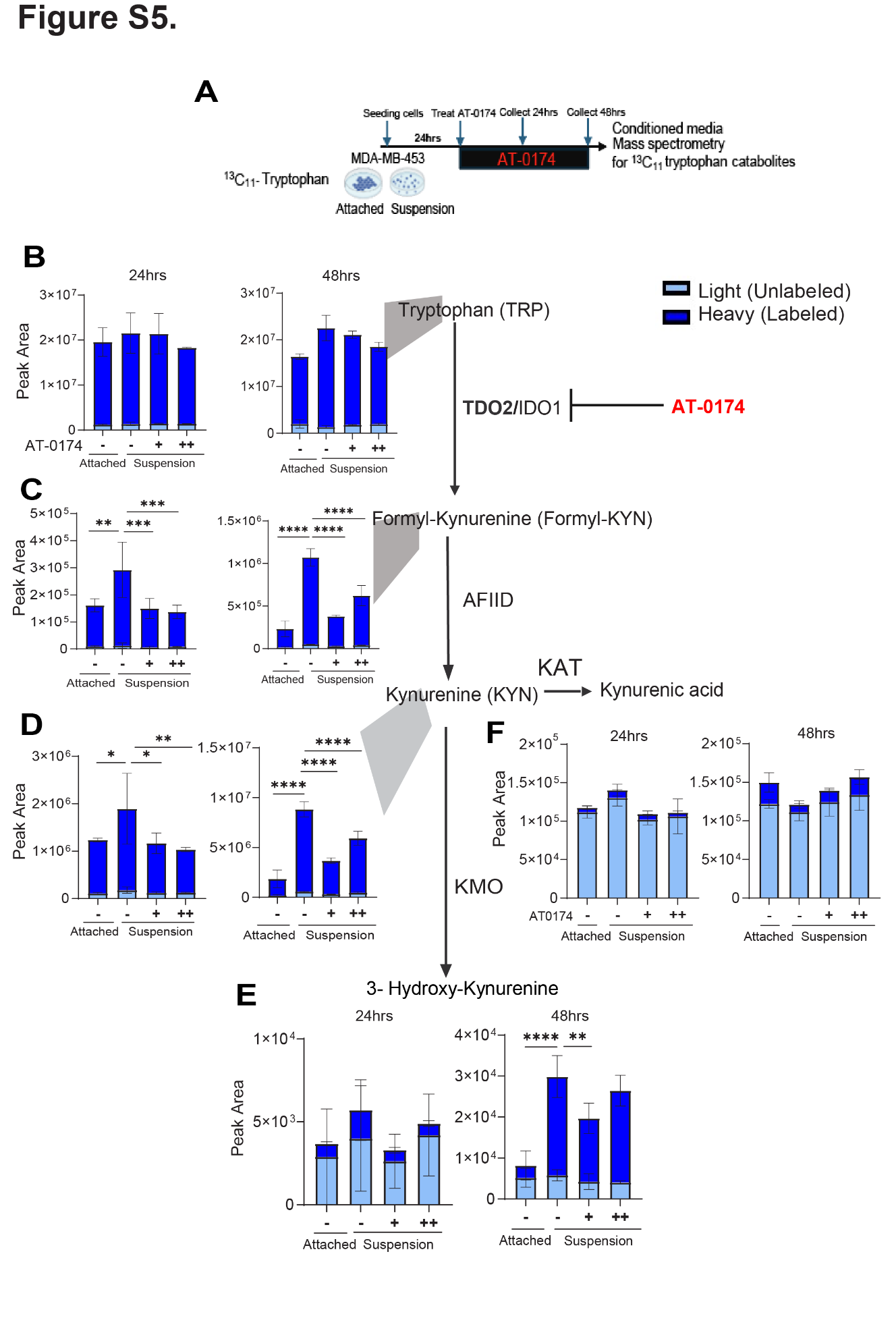


**Supplementary Figure S5. AT-0174 significantly decreased extracellular tryptophan catabolites produced by MDA-MBA-453 cells.**A. Schematic of the study design: cells in attached versus forced suspension culture were incubated in media with (^13^C_11_) labeled tryptophan (TRP) for 24 hours then cells were treated with vehicle control (DMSO) or 1μM (“+”) or 10μM AT0174 (“++”) for 24 or 48 hour. Conditioned media was analyzed by mass spectrometry. B.^13^C_11_-labeled TRP and downstream catabolites, C. Formyl-Kynurenine (Formyl-KYN), D. Kynurenine (KYN), E. 3-Hydro-Kynurenine and F. Kynurenic acid were measured in media and fractions represented as peak area. Biological replicates (n=3) were conducted in each group, and the data displayed as Mean ± SEM with 2-way ANOVA analysis * p<0.05, **p<0.01, **p<0.001, ****p<0.0001.
