# Supplementary material for: Blocking Tryptophan Catabolism Reduces Triple-Negative Breast Cancer Invasive Capacity
Source: Cancer Res Commun. 2024 Oct 16;4(10):2699–713. doi: 10.1158/2767-9764.CRC-24-0272 (PMC11484926; doi:10.1158/2767-9764.CRC-24-0272)
Supplement: Supplementary Figure S6 — Pharmacologic inhibition or genetic knockdown of TDO2 reduces TNBC anchorage independent growth. [file crc-24-0272_supplementary_figure_s6_suppsf6.docx]

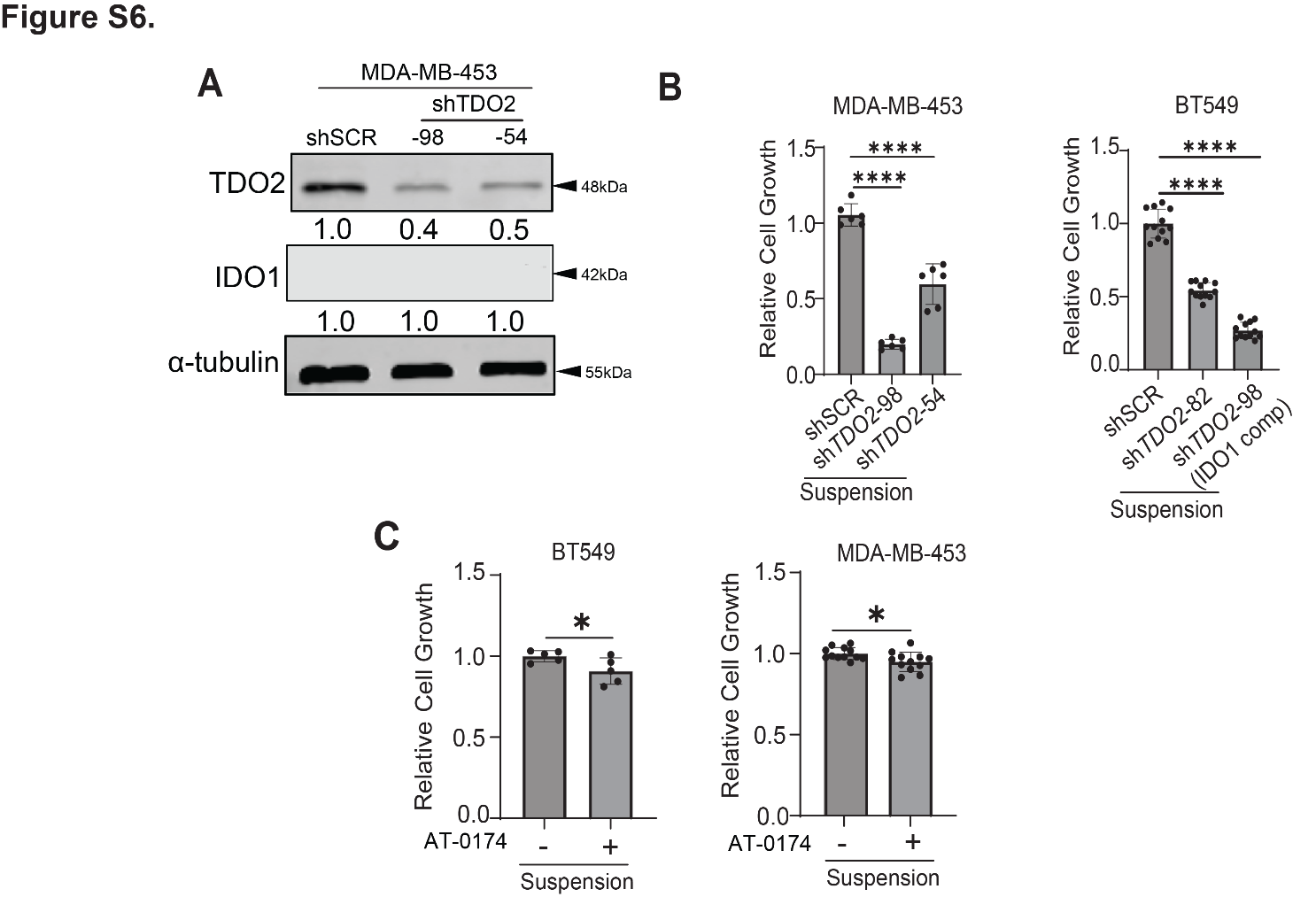


**Supplementary Figure S6.** **Pharmacologic inhibition or genetic knockdown of TDO2 reduces TNBC anchorage independent growth.** A. Immunoblot for stable scrambled control (shSCR) and *TDO2* knockdown (sh*TDO2*) in MDA-MB-453. B. BT549 and MDA-MB-453 with shSCR or sh*TDO2* were cultured in suspension condition. C. BT549, MDA-MB-453 were cultured in suspension condition following 10μM AT-0174 treatment. The cell proliferation was measured on Day 7 by using Celltiter Glo. Mean± SD with One-way ANOVA or t-test analysis *: p<0.05, **p<0.01, ***p<0.001, ****p<0.0001.
