# Supplementary material for: Blocking Tryptophan Catabolism Reduces Triple-Negative Breast Cancer Invasive Capacity
Source: Cancer Res Commun. 2024 Oct 16;4(10):2699–713. doi: 10.1158/2767-9764.CRC-24-0272 (PMC11484926; doi:10.1158/2767-9764.CRC-24-0272)
Supplement: Supplementary Figure S7 — AT-0174 reduced invasion in TDO2 OE cells. [file crc-24-0272_supplementary_figure_s7_suppsf7.docx]

**
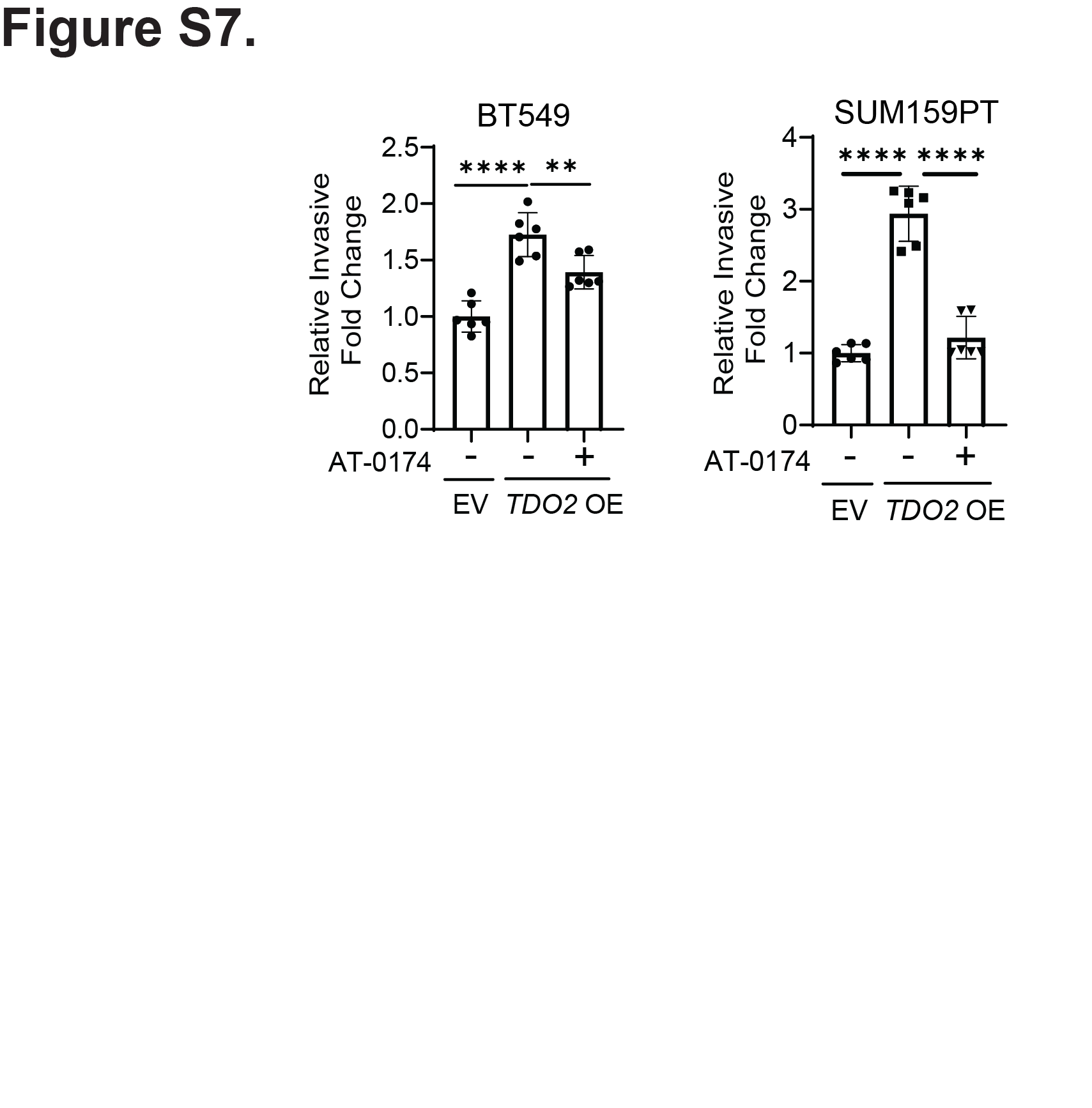
**

**Supplementary Figure S7. AT-0174 reduced invasion in *TDO2* OE cells.** A. Invasion of BT549 and SUM159PT with TDO2 overexpression (*TDO2* OE) treated with 10μM AT-0174 and empty vector (EV) control. Transwell invasion through Cultrex was assayed for 24 hours, invaded cells were stained with 0.5%/25% (v/v) crystal violets/methanol and dissolved in 10% acetic acid. Absorbance was read at the wavelength of 570nm. Mean± SD with One-way ANOVA analysis *: p<0.05, **p<0.01, ***p<0.001, ****p<0.0001.
