# Supplementary material for: Blocking Tryptophan Catabolism Reduces Triple-Negative Breast Cancer Invasive Capacity
Source: Cancer Res Commun. 2024 Oct 16;4(10):2699–713. doi: 10.1158/2767-9764.CRC-24-0272 (PMC11484926; doi:10.1158/2767-9764.CRC-24-0272)
Supplement: Supplementary Figure S8 — Treatment of AT-0174, epacadostat or StemRegenin reduces invasion in SUM159PT cells. [file crc-24-0272_supplementary_figure_s8_suppsf8.docx]

**
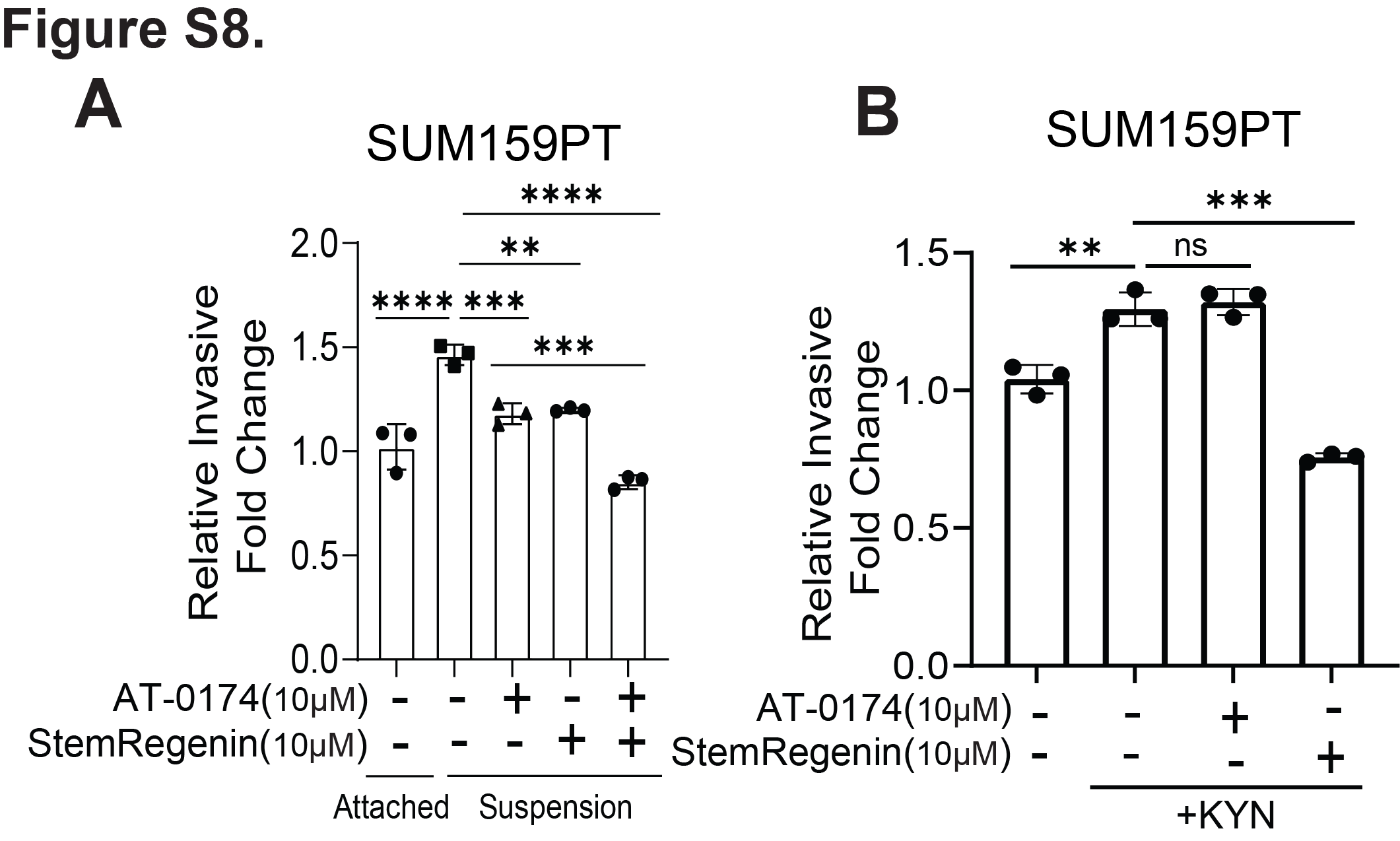
**

**Supplementary Figure S8.** **Treatment of AT-0174, epacadostat or StemRegenin reduces invasion in SUM159PT cells.** Invasion of A.SUM159PT under attached and suspension culture for 24hrs and treated with vehicle (DMSO) control, 10μM AT-0174 or 10μM StemRegenin for additional 48hrs. B. SUM159PT were treated with 10μM KYN along with AT-0174 or StemRegenin. Transwell invasion through Cultrex for 24 hours, invaded cells were stained with 0.5%/25% (v/v) crystal violets/methanol and dissolved in 10% acetic acid. Absorbance was read at the wavelength of 570nm. Mean± SD with One-way ANOVA analysis *: p<0.05, **p<0.01, ***p<0.001, ****p<0.0001.
