# Supplementary material for: Blocking Tryptophan Catabolism Reduces Triple-Negative Breast Cancer Invasive Capacity
Source: Cancer Res Commun. 2024 Oct 16;4(10):2699–713. doi: 10.1158/2767-9764.CRC-24-0272 (PMC11484926; doi:10.1158/2767-9764.CRC-24-0272)
Supplement: Supplementary Figure S9 — The AhR ChIP seq dataset shows high enrichments at the promoter region of ZEB1 transcript. [file crc-24-0272_supplementary_figure_s9_suppsf9.docx]

**
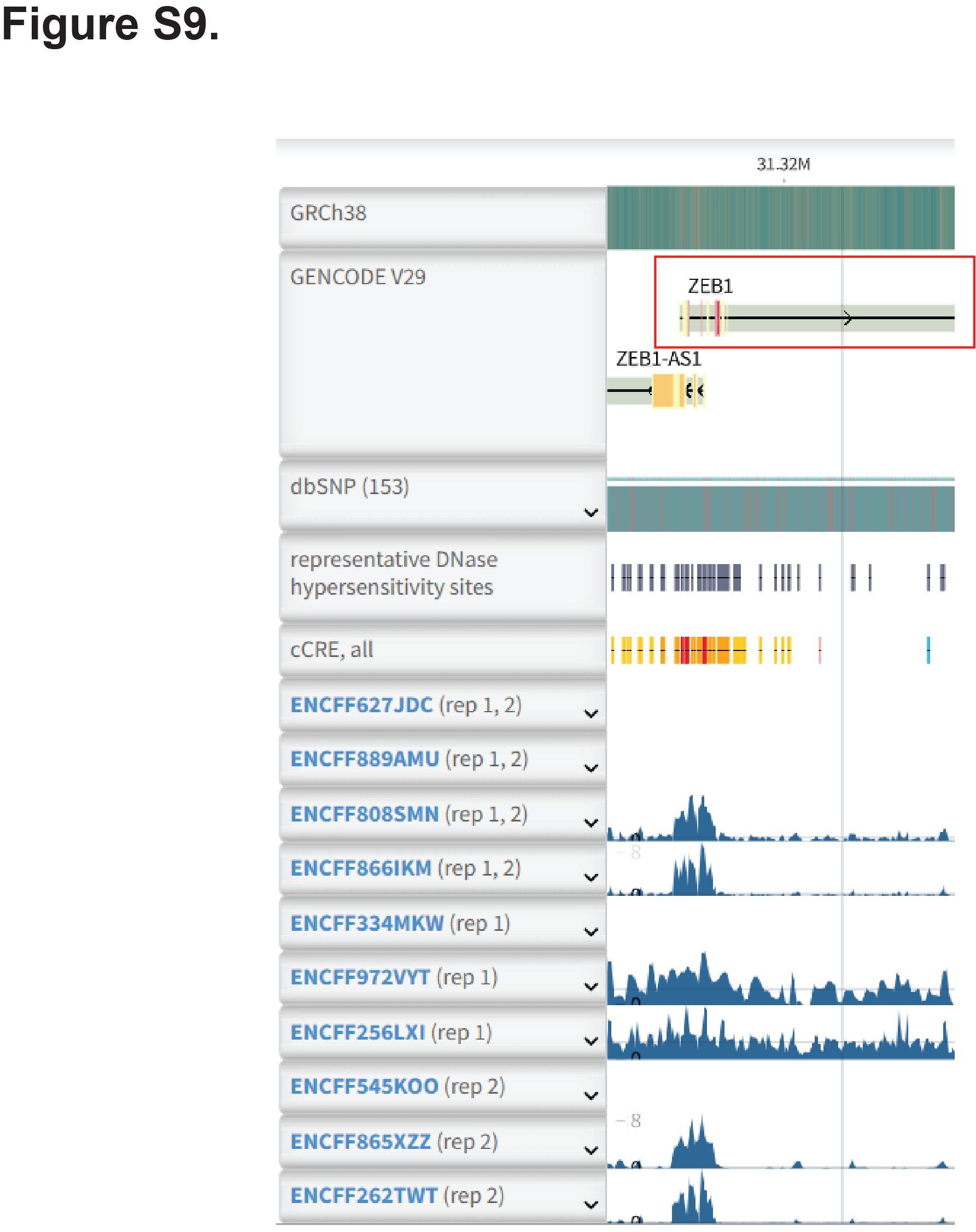
**

**Supplementary Figure S9.** **The AhR ChIP seq dataset shows high enrichments at the promoter region of *ZEB1* transcript.** AhR ChIP was performed on HepG2 cells. The public dataset (GSE127649) was extracted from the ENCODE platform.
