# Supplementary material for: Blocking Tryptophan Catabolism Reduces Triple-Negative Breast Cancer Invasive Capacity
Source: Cancer Res Commun. 2024 Oct 16;4(10):2699–713. doi: 10.1158/2767-9764.CRC-24-0272 (PMC11484926; doi:10.1158/2767-9764.CRC-24-0272)
Supplement: Supplementary Figure S10 — Genetic knockdown or pharmacological inhibition of TDO2 or AhR decreased ZEB1 mRNA expression but increased while IDO1 compensated; KYN treatment increased ZEB1 expression. [file crc-24-0272_supplementary_figure_s10_suppsf10.docx]

**
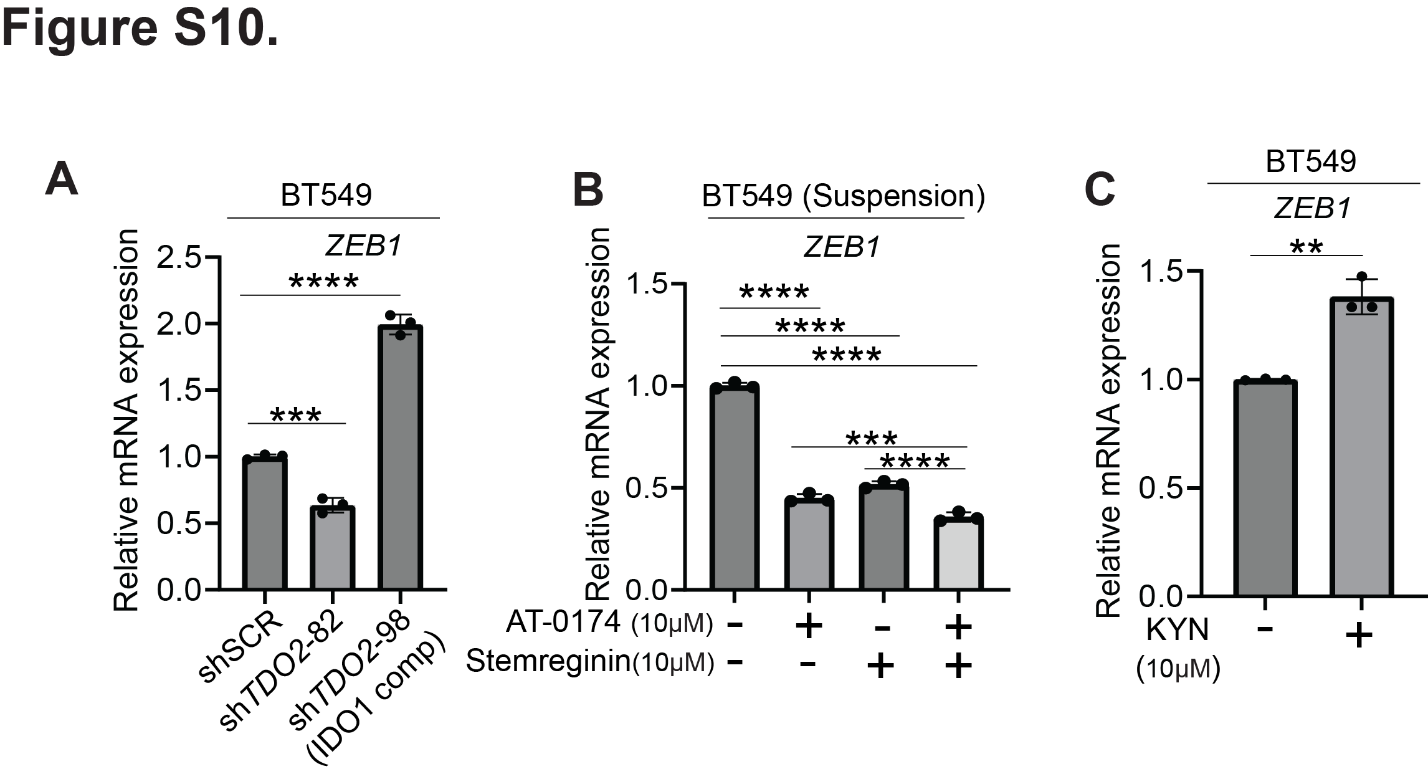
**

**Supplementary Figure S10.**  **Genetic knockdown or pharmacological inhibition of TDO2 or AhR decreased *ZEB1* mRNA expression but increased while IDO1 compensated; KYN treatment increased *ZEB1* expression.**  mRNA expression of A. BT549 with TDO2 shRNA. B. BT549 under suspension culture for 24hrs and treated with vehicle (DMSO) control, 10μM AT-0174, 10μM Stemreginin or combination for 48hrs. C. BT549 were treated with vehicle or 10μM KYN. GAPDH was used as an internal control. Mean± SD with One-way ANOVA analysis *: p<0.05, **p<0.01, ***p<0.001, ****p<0.0001.
