# Supplementary material for: Blocking Tryptophan Catabolism Reduces Triple-Negative Breast Cancer Invasive Capacity
Source: Cancer Res Commun. 2024 Oct 16;4(10):2699–713. doi: 10.1158/2767-9764.CRC-24-0272 (PMC11484926; doi:10.1158/2767-9764.CRC-24-0272)
Supplement: Supplementary Figure S11 — AhR, ZEB1 and TDO2 expression in clinical breast cancer database. [file crc-24-0272_supplementary_figure_s11_suppsf11.docx]

**
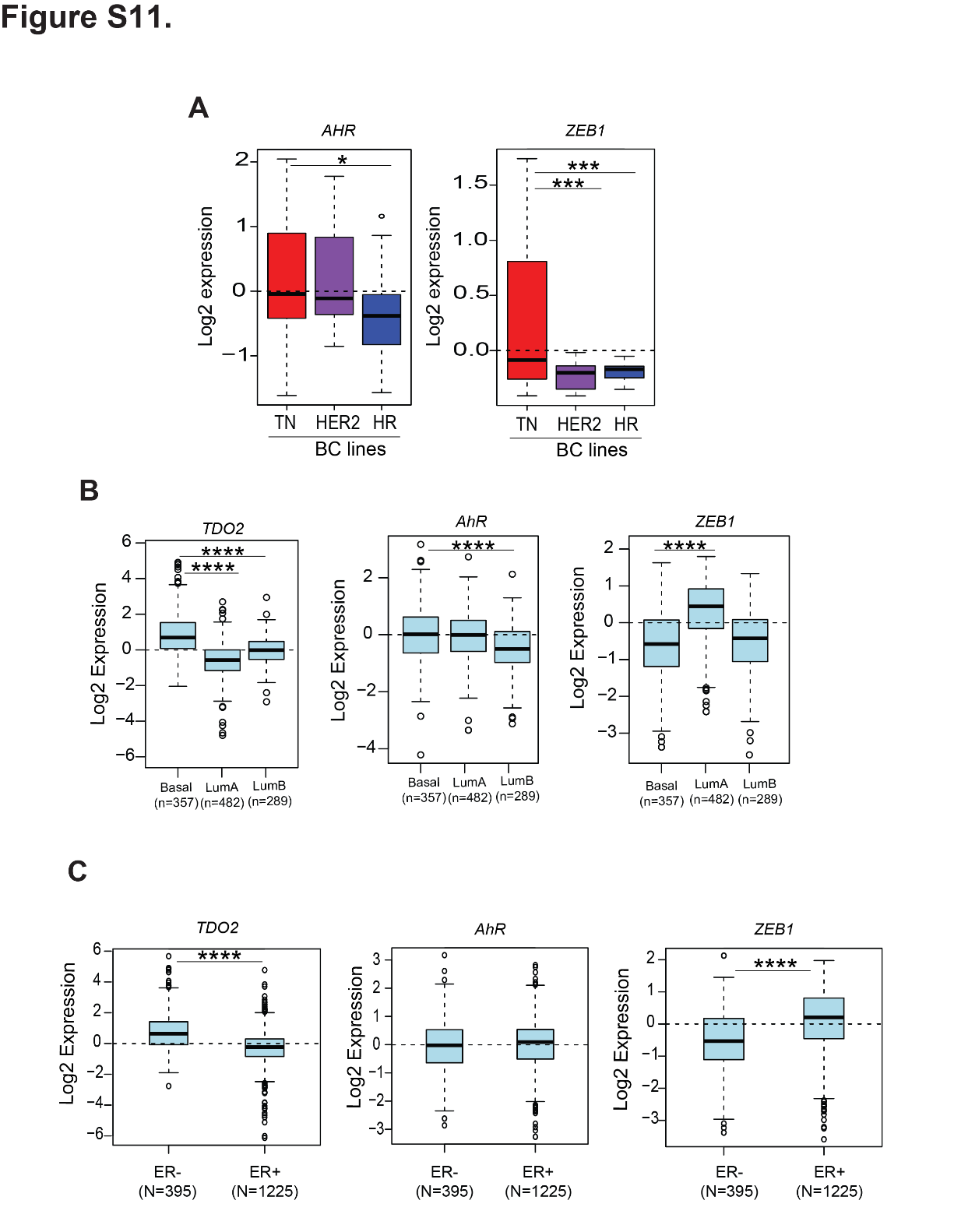
**

**Supplementary Figure S11. *AhR, ZEB1* and *TDO2* expression in clinical breast cancer database.** A. *AhR* and *ZEB1* expression in TNBC, HER2 enriched and HR+ breast cancer cell lines (n=51) B. Expression of *TDO2*, *AhR,* and *ZEB1* from patients with basal-like, Luminal A/B breast cancers. C. *TDO2, AhR,* and *ZEB1* from patients stratified with ER+/-. Gene expression-based Outcome (GOBO) database and analyzed by one-way ANOVA or t-test *: p<0.05, **p<0.01, ***p<0.001, ****p<0.0001.
